# Activation of FXR and inhibition of EZH2 synergistically inhibit colorectal cancer through cooperatively accelerating FXR nuclear location and upregulating CDX2 expression

**DOI:** 10.1038/s41419-022-04745-5

**Published:** 2022-04-21

**Authors:** Junhui Yu, Kui Yang, Jianbao Zheng, Pengwei Zhao, Jie Xia, Xuejun Sun, Wei Zhao

**Affiliations:** 1 Department of General Surgery, First Affiliated Hospital of Xi’an Jiaotong University, 710061 Xi’an, PR China; 2 State Key Laboratory of Bioactive Substance and Function of Natural Medicines, Department of New Drug Research and Development, Institute of Materia Medica, Chinese Academy of Medical Sciences and Peking Union Medical College, 100050 Beijing, PR China

**Keywords:** Colon cancer, Target identification, Targeted therapies

## Abstract

Our previous study indicated that colon cancer cells varied in sensitivity to pharmacological farnesoid X receptor (FXR) activation. Herein, we explore the regulatory mechanism of FXR in colorectal cancer (CRC) development and aim to design effective strategies of combined treatment based on the regulatory axis. We found that the expression of FXR was negatively correlated with enhancer of zeste homolog 2 (EZH2) in colon cancer tissues. EZH2 transcriptionally suppressed FXR via H3K27me3. The combination of FXR agonist OCA plus EZH2 inhibitor GSK126 acted in a synergistic manner across four colon cancer cells, efficiently inhibiting clonogenic growth and invasion in vitro, retarding tumor growth in vivo, preventing the G0/G1 to S phase transition, and inducing caspase-dependent apoptosis. Benign control cells FHC were growth-arrested without apoptosis induction, but retained long-term proliferation and invasion capacity. Mechanistically, the drug combination dramatically accelerated FXR nuclear location and cooperatively upregulated caudal-related homeobox transcription factor 2 (CDX2) expression. The depletion of CDX2 antagonized the synergistic effects of the drug combination on tumor inhibition. In conclusion, our study demonstrated histone modification-mediated FXR silencing by EZH2 in colorectal tumorigenesis, which offers useful evidence for the clinical use of FXR agonists combined with EZH2 inhibitors in combating CRC.

## Introduction

Colorectal cancer (CRC) ranks the third commonly diagnosed cancer globally, which caused approximately 935,000 death every year. Most CRC patients are diagnosed at the advanced stage due to insidious symptoms in the early stage [1, 2]. Despite the great progress has been achieved in multimodality therapy for CRC, the prognosis of advanced-stage CRC is still poor. The five-year survival rate of stage IV CRC patients is slightly higher than 10% [3]. Hence, clarification of pivotal genes and their regulatory mechanisms during colorectal tumorigenesis is urgently required for developing innovative and effective strategies in confronting CRC.

CRC is a multifactorial disease involving both hereditary and environmental factors.

Multiple risk factors for CRC include obesity, physical inactivity, poor diets, alcohol drinking, and smoking [4]. More importantly, many mutations in genes, e.g., APC, TP53, SMAD4, PIK3CA, and KRAS, copy-number changes, chromosomal translocation patterns, and myc-directed transcriptional activation or repression contribute to colorectal tumorigenesis [5]. The complex and heterogeneous genetic characteristics of CRC hinder the discovery of universal molecular targets, and the effectiveness of subsequent targeted therapies.

Epidemiological studies, animal models, and clinical studies identify fecal bile acid as a risk factor for colon cancer [6]. High levels of bile acids elicit hazardous influence on colonic mucosa characterized by DNA oxidative damage [7], inflammation [8], and hyperproliferation [9]. Farnesoid X receptor (FXR, translated by the NR1H4 gene), a bile acid-activated nuclear receptor, regulates the expression of target genes involved in lipid, cholesterol, and glucose metabolism [10]. Emerging evidence supports the pivot role of FXR in tumorigenesis. The deficiency of FXR correlates with tumor progression and often signifies adverse clinical outcomes [11]. Recently, obeticholic acid (OCA), a novel FXR agonist, preliminarily exhibits a tumor inhibitory activity in CRC [12], hepatocellular carcinoma (HCC) [13], and cholangiocarcinoma [14]. However, the complex regulatory network of FXR in tumor might hamper the implementation of FXR agonist in clinical treatment [15]. We previously demonstrated that the antiparasitic drug nitazoxanide (NTZ) acted synergistically with OCA in tumor inhibition by abrogating β-Catenin expression [16]. GW4064, an FXR agonist, combined with acyclic retinoid (ACR), exerted synergistic inhibitory effects on the growth of HCC with lower doses of both agents [17]. Generally, epigenetic aberrations including DNA methylation, chromatin remodeling, and histone modification contribute to tumorigenesis by dysregulating the transcriptional activity [18]. Herein, the epigenetic regulatory mechanism of FXR in colorectal tumorigenesis is deeply explored.

Enhancer of zeste homolog 2 (EZH2), a member of polycomb-repressive complex 2 (PRC2) complex, regulates the expression of target genes by catalyzing trimethylation of lysine 27 on histone H3 (H3K27me3) [19]. Due to the selectivity of PRC2-mediated gene silencing, EZH2 functions oncogenic or tumor-suppressive role in human malignancies. In CRC tissues, EZH2 expression is significantly elevated and often correlates with poor prognosis [20, 21]. Importantly, targeting EZH2 could significantly enhance death receptors and reduce tumorigenic potential of CRC in vivo and in vitro [22], supporting EZH2 as a promising therapeutic targe.

In the present study, we aim to explore the epigenetic modification of FXR in CRC, and design innovative and effective strategies of combined treatment based on the identified regulatory axis

## Materials and methods

### Cell cultures

Colon cancer cells HT-29, HCT116, SW403, SW480, SW620, RKO, HT-29, and DLD-1 and normal colon epithelial cells FHC (Shanghai Institute of Cell Biology, Chinese Academy of Sciences) were all routinely cultured in DMEM supplemented with 10% fetal bovine serum (FBS) at 5% CO_2_ at 37 °C. Once the cells reached 70% confluence, they were treated with various doses of OCA for 48 h along with GSK126.

### Bioinfiomatic analysis

Bioinfiomatic analysis is conducted via the TCGA database and NCBI Gene Expression Omnibus (GEO, https://www.ncbi.nlm.nih.gov/gds/) under accession numbers (GSE8023)

### Lentiviral vectors and transfection

Lentiviral vectors with EZH2/FXR shRNA or overexpression were purchased from GeneChem Co., Ltd. (Shanghai, China). All transfections were performed according to the manufacturer’s instructions.

### Drug combination studies

Cells per well were seeded in the 96-well plates at a density of 5 × 10^3^, and then treated with a single compound or with a combination of OCA and GSK126 for another 48 h. Cell viability was measured using the CCK-8 assay [23]. Combination index (CI) and fraction affected (Fa) values were calculated using Compusyn software. CI > 1 indicates antagonism, CI = 1 indicates an additive effect, and CI < 1 indicates synergy.

### CCK8, colony formation, cell cycle, and apoptosis assays

CCK8 assays were performed as described previously [23]. For colony formation assay, three hundred cells were seeded and cultured for 14 days. Colonies (≥50 cells/colony) were counted. Cell cycle distributions were evaluated by flow cytometry as previously described [23]. For apoptosis assay, cells were labeled with Annexin V PE/7-AAD (BD Biosciences, Franklin Lakes, NJ, USA) according to the manufacturer’s protocol as previously described [24]. Each experiment was performed in triplicate.

### Wound-healing assays

Cells were cultured in six-well plates until 90% confluence. Pipette tips (10 μL) were then utilized to scratch artificial vertical lines. The cells were maintained in FBS-free medium for an additional 48 h. The images of wound closure were captured under a microscope at 0 and 48 h.

### Transwell assays

Cell invasion was measured by using Transwell plates (Corning, New York, NY, USA) with Matrigel (BD, Franklin Lakes, NJ, USA). The lower chamber was filled with 600 μL of DMEM containing 20% FBS. The upper chamber filters were pre-coated with 50 µL of Matrigel and plated at 10 × 10^4^ cells per upper chamber. The cells were incubated at 37 °C for 48 h. After incubation, non-migratory cells on the upper surface of the Transwell inserts were removed by washing with fresh phosphate buffered solution (PBS). The migratory or invading cells on the underside of the membrane were fixed with 4% paraformaldehyde and stained with 1% crystal violet. The number of cells was counted in three randomly selected fields of fixed cells under an inverted microscope. Each experiment was performed in triplicate.

### Nude mouse xenograft assay

All animal experiments were performed in accordance with the institutional guidelines, and were approved by the Laboratory Animal Center of Xi’an Jiaotong University. The 5 week-old female BALB/c-nude mice was purchased from Shanghai SLAC Laboratory Animal Co., Ltd. (Shanghai, China). The mice were injected with 5 × 10^6^ colon cancer cells into the right flanks to establish xenograft tumor model. Once the size of xenograft tumors reached approximately 100 mm^3^, the nude mice were randomly divided into four subgroups, each consisting of five mice and were administered by oral gavage of OCA (10 mg/kg/day) and GSK126 (50 mg/kg/day) alone or in combination for consecutive 18 days. Tumor size was monitored using callipers every 3 days, and the tumor volume was measured the according to the formula (*a* × *b*^2^ × 0.5, *a*: length, *b*: width). After 18 days of drug administration, the mice were executed and the xenograft tumors were isolated and weighted.

### RNA isolation and real-time PCR

RNA isolation, complementary DNA (cDNA) synthesized, and real-time PCR were performed as described previously [23]. The sequences of primers were summarized in Supplementary Table 1. Each experiment was performed in triplicate.

### Immunohistochemistry (IHC)

The protocol was performed as previously described [23]. Briefly, the extent of stained cells (0, 0–5%; 1, 6–25%; 2, 26–50%; 3, 51–75%; and 4, 76–100%) and the staining intensity (0, negative; 1, light brown; 2, brown; and 3, dark brown) were recorded. The immunoreactivity scores (IRSs) were defined as the product of extent and intensity scores. An IRS of >3 was considered as positive expression.

### Total protein extraction and Western blot

The detailed protocol was performed as described previously [23]. The antibody information was presented in Supplementary Table 2. Each experiment was performed in triplicate.

### Immunofluorescence (IF)

The cells were fixed with 4% paraformaldehyde for 20 min and permeabilized with 0.2% Triton X-100 for 10 min. After blocking with 5% bovine serum albumin (BSA) for 30 min at room temperature, the cells were incubated at 4 °C overnight with primary antibodies against FXR or β-Catenin (1:100 dilution). The dishes were washed three times with PBS for 10 min each and then incubated with Alexa Fluor 594/488-conjugated secondary antibodies (1:400 dilution, Invitrogen, Carlsbad, CA, USA) for 1 h at room temperature. The nuclei were stained with DAPI (10 mg/ml) for 10 min. The samples were examined via microscopy (Leica Microsystems, Heidelberg, Germany) to analyze the subcellular localization of FXR and β-Catenin.

### Luciferase reporter assay

For promoter analyses, a fragment of the CDX2 5′-flanking sequence (from −1059 bp to +89 bp) and other truncated fragments were cloned into the pGL3.0 Basic Vector (Promega, Madison, WI, USA) to generate a CDX2 full promoter reporter construct and the truncated ones (Supplementary Table 1). The plasmids containing firefly luciferase reporters of CDX2 promoter and the truncated ones, and the pTK-RL plasmids were co-transfected into cells. The detailed protocol was carried out as described previously [23].

### Quantitative chromatin immunoprecipitation (qChIP)

Cells were subjected to ChIP using the EZ-ChIP Kit (Millipore, Bedford, MA, USA).

The detailed protocol was performed as described previously [23]. Real-time PCR was conducted to amplify the regions of DNA fragments by using special primers (Supplementary Table 1). Each experiment was performed in triplicate.

### Statistical analysis

The differences among the groups were compared by the Student’s *t*-test or one-way ANOVA. All statistical analyses were performed using the SPSS statistical package (SPSS Inc., Chicago, IL, USA). *P* < 0.05 was considered statistically significant.

## Results

### FXR expression is transcriptionally suppressed by EZH2 via H3K27me3

**Fig. 1:**
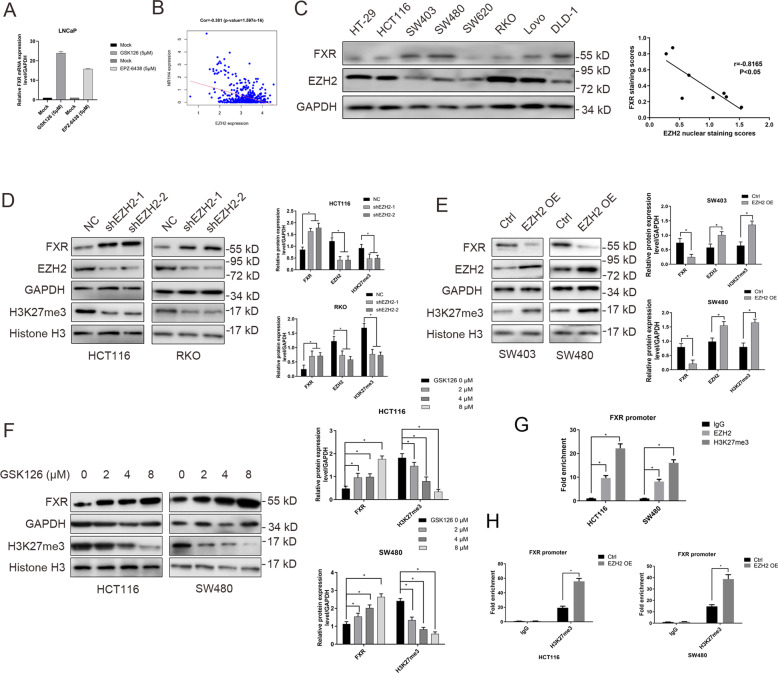
EZH2 negatively regulated the expression of FXR by H3K27me3. **a** Relative NR1H4 mRNA levels in LNCaP cells treated or not with GSK126. **b** Correlation analysis of EZH2 and FXR mRNA level in colon cancer tissues from TCGA database. **c** The protein levels of EZH2 and FXR in colon cancer cells by western blotting analysis (left panel: gel bands; right panel: correlation analysis between EZH2 and FXR). **d** The protein levels of FXR and H3K27me3 in HCT116 and RKO cells with EZH2 depletion by western blotting analysis (left panel: gel bands; right panel: quantitative analysis of these proteins). **e** The protein levels of FXR and H3K27me3 in SW403 and SW480 cells with EZH2 overexpression by western blotting analysis (left panel: gel bands; right panel: quantitative analysis of these proteins). **f** The effect of EZH2 inhibitor GSK126 on the protein levels of FXR and H3K27me3 in SW403 and SW480 cells by western blotting analysis (left panel: gel bands; right panel: quantitative analysis of these proteins). **g** The ChIP analysis of the enrichment of H3K27me3 and EZH2 in the FXR promoter region. **h** The effect of enhancing EZH2 expression on the enrichment of H3K27me3 to in the FXR promoter region. All data are the mean ± SD of three independent experiments. *P < 0.05.

Previous studies indicated that FXR deficiency confers poor prognosis of patients with several cancer types [11, 14, 25], which was further confirmed in colon cancer patients in our previous study [26]. Restoration of FXR significantly suppressed aggressive behavior of colon cancer [26]. The pivotal role of FXR in CRC onset highlights the importance to elucidate the regulatory mechanisms of FXR. Intriguingly, analysis of GEO database (GSE8023) revealed that treatment of LnCaP cells with EZH2 inhibitor GSK126 or EPZ-6438 strikingly induced FXR mRNA up-regulation (Fig. 1a). TCGA database mining also supported an inverse correlation between EZH2 and FXR in the patients with colon cancer (Fig. 1b). Hence, we inferred that EZH2 negatively regulated FXR expression in CRC.

We firstly analyzed the protein levels of FXR and EZH2 in eight colon cancer cells by western blotting. FXR is expressed in colon cancer cells at variable levels; however, an inverse correlation was observed between FXR and EZH2 expression (Fig. 1c). HCT116 and RKO cells harboring high EZH2 level were conducted with EZH2 knockdown and SW403 and SW480 with EZH2 deficiency were conducted with EZH2 overexpression. Transfection with EZH2 shRNA lentiviral dramatically increased the levels of FXR in HCT116 and RKO cells (Fig. 1d), while enhancing EZH2 expression led to a decreased level of FXR in SW403 and SW480 cells (Fig. 1e). As expected, GSK126 strikingly elevated the expression of FXR in HCT116 and SW480 cells (Fig. 1f). The analysis from qChIP assay revealed an enrichment of H3K27me3 and EZH2 in the promoter region of FXR (Fig. 1g). Moreover, ectopic expression of EZH2 enhanced the binding of H3K27me3 to FXR upstream region (Fig. 1h).

**Fig. 2:**
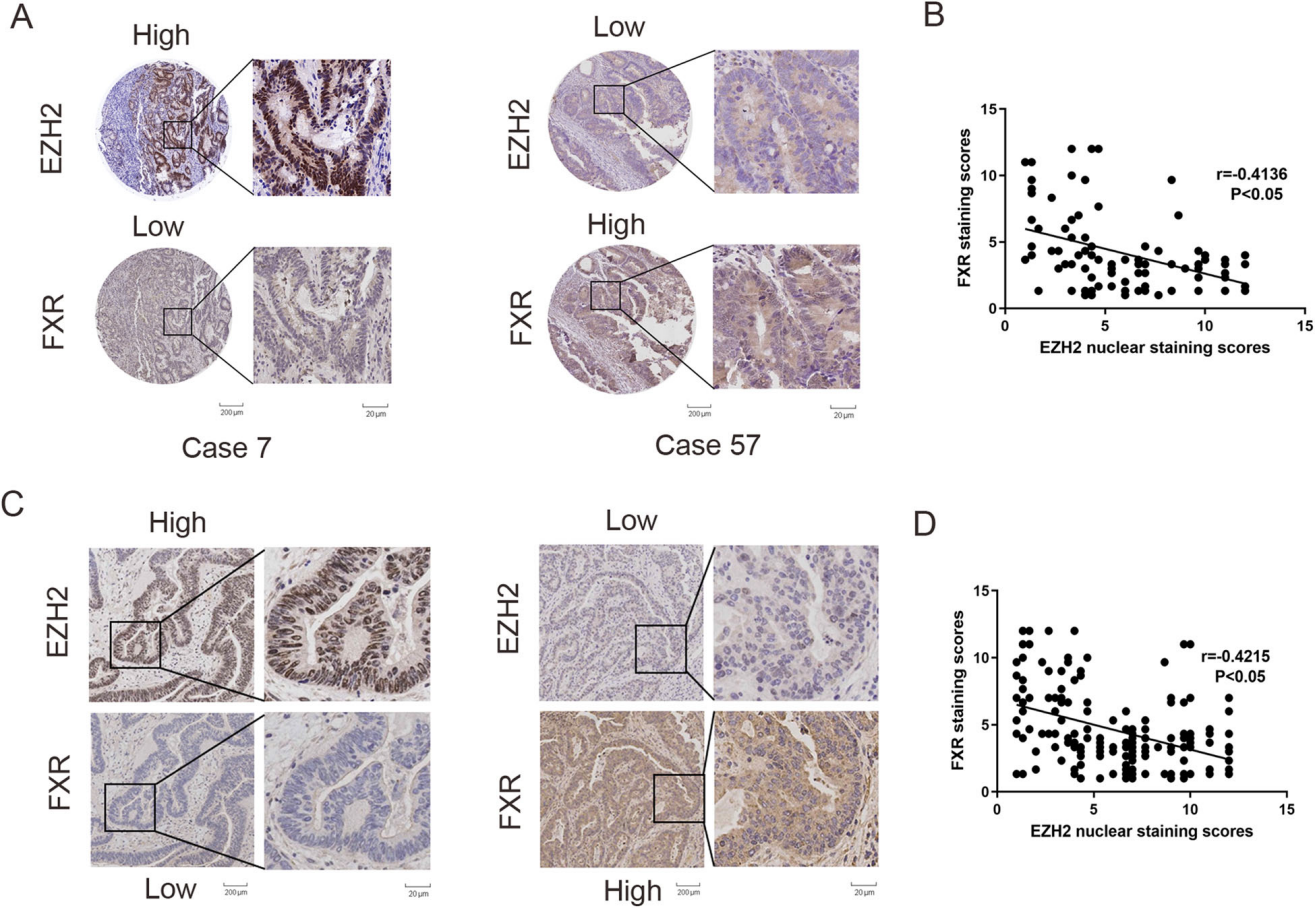
An inverse correlation between EZH2 and FXR was found in the patients with colon cancer. **a** EZH2 and FXR expression in tissue microarrays purchased from Shaanxi Kexin Biotechnology Co., Ltd, including 90 pairs of CRC samples and paired NC tissues by IHC staining (cohort I). **b** Correlation analysis of EZH2 and FXR in CRC tissue microarrays. **c** EZH2 and FXR expression in an independent cohort of 403 colon cancer tissues sample from at the First Affiliated Hospital of Xi’an Jiaotong University by IHC staining (cohort II). **d** Correlation analysis of EZH2 and FXR in 403 colon cancer tissues microarrays. All data are the mean ± SD of three independent experiments. *P < 0.05.

The clinical correlation between EZH2 and FXR in CRC was explored in the tissue microarrays containing 90 pairs of colon cancer by IHC staining (cohort I, Fig. 2a). The EZH2 level was negatively correlated with FXR (Fig. 2b). Moreover, another independent cohort of 162 colon cancer patients revealed a negative correlation between FXR and EZH2 (Fig. 2c, d). Altogether, these results suggested that EZH2 acts a negative regulator of FXR in CRC through H3K27me3.

### FXR transcriptionally activates the expression of tumor suppressor CDX2

We next explored the downstream targets directly regulated by FXR in CRC. Our previous study revealed the regulatory effect of FXR on caudal-related homeobox transcription factor 2 (CDX2), a tumor suppressor, although the precise mechanism is unclear [27]. Intriguingly, bioinformatic analysis of the proximal promoter of CDX2 identified a potential FXR response element, an inverted repeat-1 (IR-1, 5′-AGGTCGCTGCCCT-3′), located −449 to −461 bp upstream of the transcription start site. Hence, we attempted to validate the involvement of CDX2 in FXR-mediated tumor inhibition in CRC. Firstly, CDX2 promoter reporter constructs with full length from −1059 bp to +89 bp or truncated promoter were designed. The dual-luciferase reporter assay indicated that enhancing FXR expression induced an elevation of luciferase activity of constructs with the −1059, −819, or 519 bp to +89 bp region; however, deficiency of −519 bp to −376 bp region abolished this induction (Supplementary Fig. 1a). These data indicated that FXR might bind to the −519 bp to −376 bp region of CDX2 promoter to transactivate CDX2. A qChIP assay was then carried out to identify the special site of FXR binding to CDX2 promoter. This fragment corresponding to IR-1 element was enhanced upon FXR overexpression (Supplementary Fig. 1b). Meanwhile, in relation to the single drug, OCA and GSK126 combination dramatically enhanced the binding of FXR to the IR-1 element in CDX2 promoter region (Supplementary Fig. 1c).

Furthermore, ectopic expression of FXR led to an up-regulation of CDX2 levels in in SW403 and SW480 cells (Supplementary Fig. 1d, e). Conversely, FXR depletion in HCT116 and RKO cells decreased CDX2 levels (Supplementary Fig. 1f, g). Collectively, our study indicated that FXR transcriptionally activates the expression of tumor suppressor CDX2.

### Inhibition of EZH2 acts synergistically activation of FXR with in colon cancer cells

We previously reported that that colon cancer cells exhibited the varying responses to FXR agonist OCA [16]. Based on IC_50_ values, cells were clearly divided into two groups with low and high sensitivity (Supplementary Fig. 2a). IC_50_ values of RKO and HCT116 cells were below 1 μM in the high-sensitive group, but well above 4 μM in the low sensitive group with SW403 and SW480 cells. Normal colon epithelial cells FHC reacted to OCA at low concentration (IC50 0.34 μM). In the present study, the regulation of FXR by EZH2 renders us to speculate that EZH2 inhibition enhances the therapeutic effect of FXR agonist OCA against CRC.

We firstly assessed the IC50 values of colon cancer cells and FHC cells response to EZH2 inhibitor GSK126. The results indicated that GSK126 inhibited the growth of HCT116, SW403, SW480, RKO, and FHC cells with IC50 values of approximately 3.347, 6.155, 4.378, 3.718, and 0.611 μM, respectively (Supplementary Fig. 2a). The extent of synergism of GSK126 combined with OCA was analyzed by combination index (CI) from CompuSyn software. Strong synergisms were observed in four colon cancer cells (Supplementary Fig. 2c–f). Intriguingly, the strongest synergies were seen in the low OCA-sensitive SW480 and SW403 cells (Supplementary Fig. 2e, f). Conversely, a limited synergistical effect was found in normal colon epithelial cells FHC (Supplementary Fig. 2b). Altogether, this evidence suggests that EZH2 inhibitor might enhance the efficacy of FXR agonist against CRC.

### FXR agonist and EZH2 inhibitor synergistically inhibit colon cancer cell proliferation and migration

**Fig. 3:**
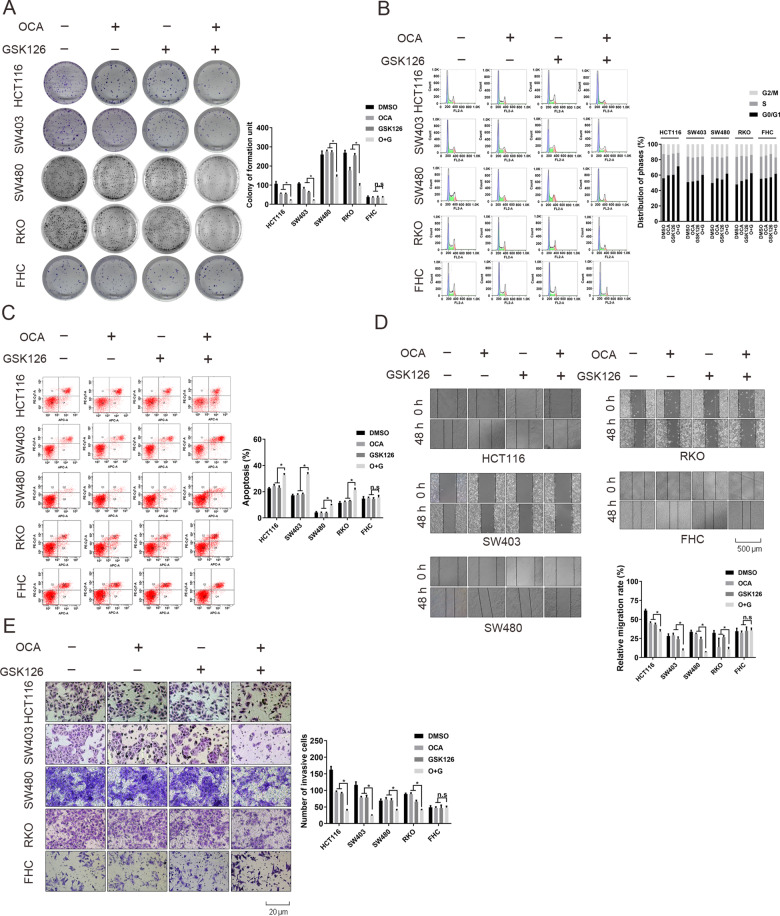
OCA and GSK126 synergistically inhibited the tumorigenic properties of colon epithelial cells in vitro. **a**–**c** The effect of OCA, GSK126 alone, or OCA plus GSK126 on colony formation (**a** left panel: colony formation assays; right panel: quantitation of the data), cell cycle distribution (**b** left panel: cell cycle analysis assays; right panel: quantitation of the data), and apoptosis (**c** left panel: apoptosis assays; right panel: quantitation of the data) of colon epithelial cells. **d**, **e** The effect of OCA, GSK126 alone or OCA plus GSK126 on the migration (**d** up panel: wound-healing assays; down panel: quantitation of the data).and invasion (**e** left panel: transwell assays; right panel: quantitation of the data) of colon epithelial cells. All data are the mean ± SD of three independent experiments. *P < 0.05.

We then carried out a series of in vitro experiments to evaluate the synergistical effect of OCA combined with GSK126. RKO, SW403, SW480, HCT116, and FHC cells were treated with OCA and GSK126 alone or in combination. Dosages of combined treatment were adopted from synergistic combinations and blow the IC_50_ of the two drugs. Firstly, in relation to the single drug, OCA and GSK126 combination consistently repressed colony-forming ability of four colon cancer cells (Fig. 3a). Notably, no remarkable clonogenic growth was seen in FHC cells. Next, we analyzed cell cycle distribution of cells exposure to OCA and GSK126 combination by flow cytometry. The combined treatment induced the proportion of cells at G0/G1 phase with the decreased proportion in S phase in four colon cancer cells (Fig. 3b). FHC cells exhibited an arrest in G0/G1 with a reduction of the S-phase and G2/M fractions. Moreover, a remarkable upregulation of the apoptosis rate was induced in four colon cancer cells, but not FHC cells, upon combined treatment (Fig. 3c). Finally, combination treatment dramatically attenuated the invasive and migratory potential of four colon cancer cells (Fig. 3d–e). No significant alteration of invasion and migration was observed in FHC cells.

Consistently, the protein levels of cell cycle- and invasion-related genes were dramatically altered upon combined treatment (Supplementary Fig. 3a, b). Altogether, this evidence suggests that EZH2 inhibition enhances the therapeutic effect of FXR agonist OCA against CRC.

### FXR agonist and EZH2 inhibitor synergistically inhibits the tumorigenesis of colon cancer cells through cooperatively accelerating FXR nuclear location and upregulating CDX2 expression

**Fig. 4:**
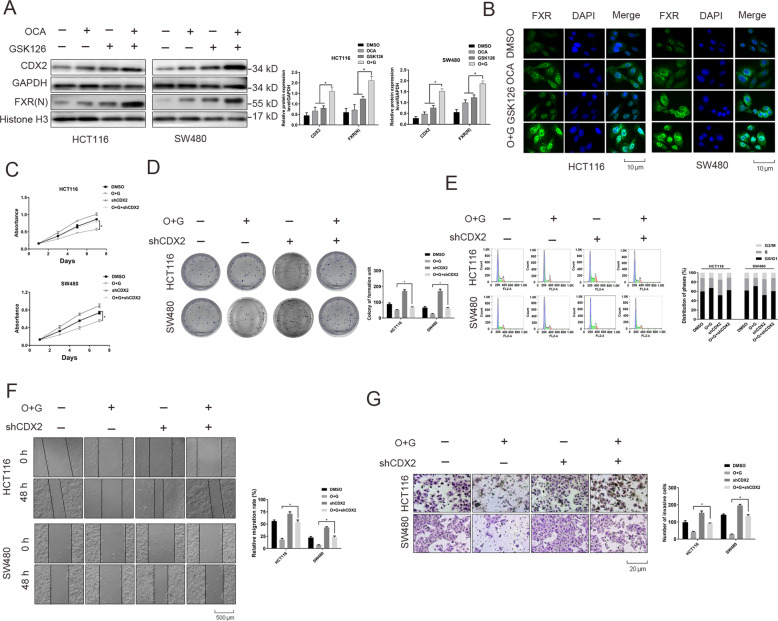
The depletion of CDX2 antagonized the synergistic effects of the combination of OCA and GSK126 on tumor inhibition. **a** The protein levels of CDX2 and FXR (nuclear) in HCT116 and SW480 cells treated with OCA and GSK126 alone or in combination by western blotting analysis (left panel: gel bands; right panel: quantitative analysis of these proteins). **b** The effect of OCA and GSK126 on the nuclear translocation of FXR detected by IF staining. **c**, **d**, **e** The effect of CDX2 depletion on the growth (**c**), colony formation (**d** left panel: colony formation assays; right panel: quantitation of the data) and cell cycle distribution (**e** left panel: cell cycle analysis assays; right panel: quantitation of the data) of HCT116 and SW480 cells treated with OCA and GSK126 alone or in combination. **f, g** The effect of CDX2 depletion on the migration (**f** left panel: wound-healing assays; right panel: quantitation of the data) and invasion (**g** left panel: transwell assays; right panel: quantitation of the data) of HCT116 and SW480 cells treated with OCA and GSK126 alone or in combination. All data are the mean ± SD of three independent experiments. *P < 0.05.

FXR agonist OCA performs its function by accelerating FXR nuclear translocation and occupancy of its target genes [28]. Meanwhile, our study found that treatment with EZH2 inhibitor GSK126 elevated the expression of FXR. Hence, we assumed that whether the synergistic inhibitory effects of GSK126 and OCA might be related to the accelerative nuclear localization of FXR. As expected, the results from western blotting analysis indicated that OCA dramatically expedited nuclear localization of FXR, which level was enhanced upon GSK126 treatment (Fig. 4a). IF assays also indicated that the combination of GSK126 and OCA expedited the nuclear localization of FXR compared to the single drug (Fig. 4b). Moreover, the role of FXR target gene, CDX2, under GSK126 and OCA treatment was investigated. In HCT116 and RKO cells, addition of GSK126 and OCA alone effectively elevated the expression of CDX2, while the effect was much stronger under the combined treatment (Fig. 4a). Importantly, CDX2 depletion greatly blocked the inhibitory effects of GSK126 and OCA on the proliferation and invasion of colon cancer cells (Fig. 4c–g).

**Fig. 5:**
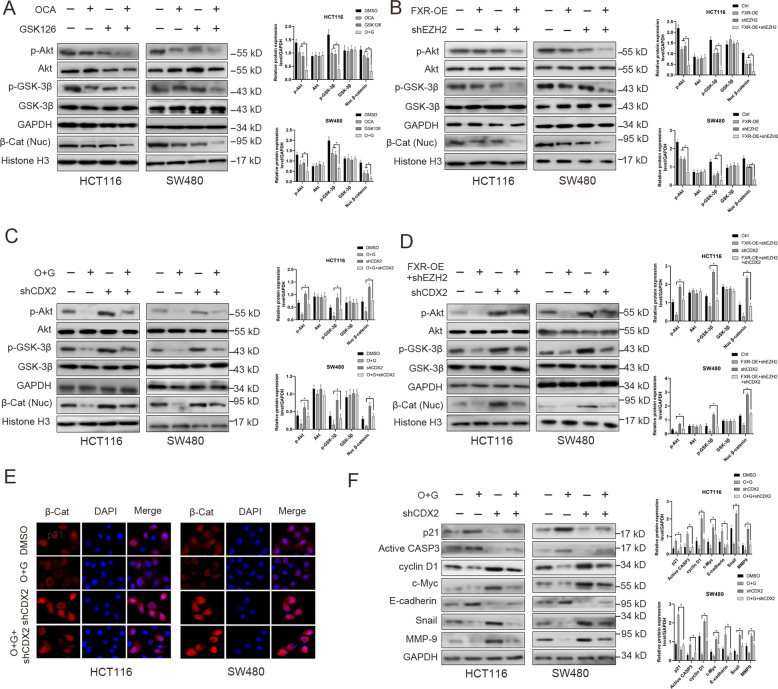
The combination of OCA and GSK126 synergistically accelerated FXR nuclear localization and promoted CDX2 expression and its regulated signaling pathways. **a** The protein levels of AKT/p-AKT (Ser473), GSK-3β/p-GSK-3β (Ser9), and β-Catenin (Nuclear) in HCT116 and SW480 cells treated with OCA and GSK126 alone or in combination by western blotting analysis (left panel: gel bands; right panel: quantitative analysis of these proteins). **b** The protein levels of AKT/p-AKT (Ser473), GSK-3β/p-GSK-3β (Ser9) and β-Catenin (Nuclear) in HCT116 and SW480 cells with FXR overexpression, EZH2 depletion alone or in combination by western blotting analysis (left panel: gel bands; right panel: quantitative analysis of these proteins). **c**, **d** The effect of CDX2 depletion on the he protein levels of AKT/p-AKT (Ser473), GSK-3β/p-GSK-3β (Ser9), and β-Catenin (Nuclear) in HCT116 and SW480 with OCA and GSK126 combination (**c** left panel: gel bands; right panel: quantitative analysis of these proteins) or FXR overexpression and EZH2 depletion (**d** left panel: gel bands; right panel: quantitative analysis of these proteins). **e** The effect of OCA and GSK126 on the nuclear translocation of β-Catenin detected by IF staining. **f** The effect of CDX2 depletion on the protein levels of cell cycle-related, apoptosis-related, and EMT-related proteins in HCT116 and SW480 cells treated with OCA and GSK126 alone or in combination by western blotting analysis (left panel: gel bands; right panel: quantitative analysis of these proteins). All data are the mean ± SD of three independent experiments. *P < 0.05.

Consequently, the signaling pathways regulated by CDX2 were further investigated when administering a combined GSK126 and OCA treatment. Our previous studies demonstrated that CDX2 retarded Akt and GSK-3β phosphorylation, and thereby inhibited β-Catenin nuclear translocation [29]. In HCT116 and SW480 cells, the combination of GSK126 and OCA synergistically retarded the phosphorylation of Akt (Ser473) and GSK-3β (Ser9) and decreased nuclear β-Catenin protein levels compared to the single drug (Fig. 5a); meanwhile, CDX2 depletion antagonized this effect under the combined treatment (Fig. 5c). Overexpression of FXR and knockdown of EZH2 achieved the comparable effects (Fig. 5b), which was abrogated by CDX2 (Fig. 5d). Furthermore, the TOP/FOP-Flash reporter assay indicated that, relative to the single drug treatment, the combination of OCA and GSK126 or overexpression of FXR and knockdown of EZH2 synergistically inhibited TCF/LEF transcriptional activities (Supplementary Fig. 4a, b), which was abrogated by CDX2 knockdown (Supplementary Fig. 4c, d). The results from IF assays indicated that the combination of GSK126 and OCA synergistically suppressed the nuclear translocation of β-Catenin, and this effect was attenuated by CDX2 depletion (Fig. 5e). Importantly, knockdown of CDX2 restored the protein levels of cell cycle- and invasion-related genes under combined treatment with OCA and GSK126 (Fig. 5f). Taken together, these data suggested that inhibition of EZH2 and activation of FXR synergistically accelerated FXR nuclear localization and promoted the expression of CDX2 and its regulated signaling pathways.

### FXR agonist and EZH2 inhibitor synergistically suppresses growth of CRC in vivo

**Fig. 6:**
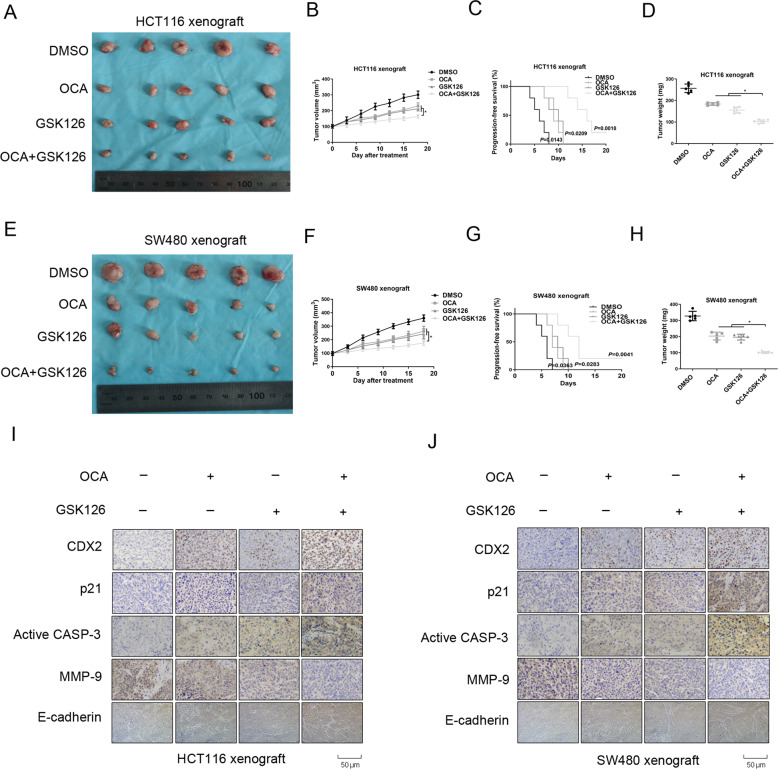
OCA and GSK126 synergistically retarded tumor growth in vivo. **a**, **e** Schematic representation of the tumor xenografts formed by HCT116 (**a**) and SW480 (**e**) cells treated with OCA, GSK126 alone or OCA plus GSK126. **b**, **d**, **f**, **h** Tumor growth curves (**b**, **f**) and tumor weights (**d**, **h**) for tumor xenograft formed by HCT116 and SW480 cells after OCA, GSK126 alone, or OCA plus GSK126 treatment. **c**, **g** Cumulative incidence plot displaying the percentage of tumor xenografts formed by HCT116 (**c**) and SW480 (**g**) cells in each treatment group that has doubled in volume as a function of time. *P* values were calculated with log-rank test. **i**, **j** IHC staining for CDX2, p21, active caspase-3, MMP-9, and E-cadherin in tumor xenografts formed by HCT116 (**i**) and SW480 (**j**) cells. *P < 0.05.

To validate the synergy of OCA and GSK126 in tumor growth suppression in vivo, nude mice with subcutaneous xenograft formed by HCT116 and SW480 cells were treated with OCA and GSK126 alone or in combination (Fig. 6a, e). Combined treatment dramatically retarded tumor growth curve and lessened tumor weight relative to the single drug treatment (Fig. 6b, d, f, h). *Q* value analyzed from Zhengjun Jin method for HCT116 and SW480 xenografts is 1.07 and 1.10, respectively [30], implying a synergistical effort of the combination treatment. Notably, the progression-free survival (PFS) of nude mice in combined treatment group is remarkably lengthened (Fig. 6c, g). Mechanistically, combination treatment results in a stronger staining of CDX2, p21, active caspase-3, and E-cadherin, and a weaker staining of MMP-9. Collectively, combination of OCA and GSK126 display strong synergy in tumor growth inhibition in vivo.

## Discussion

Chemotherapy based on fluorouracil for CRC is below expectation and limited by drug resistance and side effects. Monotargeted therapies are novel and alternative treatment options that have brought about widespread attention. However, the complicated signaling networks and tumor heterogeneity hander the implement of these drugs [31]. Recently, combination therapy has gathered tremendous interest with its ability to enhance efficacy and reduce side effects. In the present study, we demonstrated that EZH2 transcriptionally suppressed FXR expression via H3K27me3, which subsequently elevated the expression of tumor suppressor gene CDX2 and its regulatory pathways. Accordingly, the combination of EZH2 inhibitor (GSK126) and FXR agonist (OCA) synergistically elicited the antitumor activity in CRC.

FXR, a member of the nuclear receptor superfamily, is widely expressed in liver, intestine, and kidneys [32, 33]. Emerging evidence supports the involvement of FXR in human tumorigenesis. FXR plays a causative role in pancreatic [34] and esophageal [35] carcinomas. On the other hand, mice with FXR-deficiency are vulnerable to developing HCC [36]. The lack of FXR triggered intestinal inflammation and eventually promote colon tumorigenesis [11]. Conversely, restoration of FXR repressed intestinal cell growth and blocked CRC progression [37]. Mechanistically, FXR antagonized Wnt/β-Catenin signaling by forming a complex with β-Catenin [26]. The FXR/β-Catenin complex in turn hinders FXR-mediated transcriptional activation of target gene SHP [26]. Despite of the involvement of FXR in onset and progression of several types of cancer, the regulatory mechanism of its expression is obscure. Henein, we observed a negative clinical correlation between FXR and EZH2 expression in colon cancer tissues. EZH2 promotes transcriptional silencing by methylating histone methyltransferase H3K27 [38]. Increasing evidence supports a close relevance between EZH2 expression and human malignancies, for instance that of EZH2 was highly expressed in bladder [39], breast [40], endometrial [41], and prostate cancer [42] correlating with tumor progression. In this study, we revealed that FXR expression was silenced by EZH2 through H3K27m3 modification. Additionally, dysfunction of APC mediates FXR silencing through CpG hypermethylation of the FXR promoter region [11]. Collectively, the combination therapy based on targeting FXR and its regulatory mechanism could be a very rational and promising treatment of CRC patients.

CDX2, drosophila caudal-related homoeobox transcription factor, is an intestine-specific transcriptional factor implicated in the intestinal development and maintenance [43]. Several lines of evidences support that CDX2 is a tumor suppressor in CRC. The deficiency of CDX2 increases colorectal cancer susceptibility [44]. Our data revealed that enhancing CDX2 expression blocks tumor progression and retards liver metastasis in CRC [29, 45]. Mechanistically, CDX2 repressed epithelial-mesenchymal transition (EMT) in CRC by regulating Snail expression and β-Catenin stabilization via transcriptionally activating PTEN [29]. In this study, the IR-1 site was identified in the promoter fragments of CDX2. Moreover, the dual-luciferase reporter assay and qChIP assays demonstrated that FXR bound to the IR-1 site in the promoter region of CDX2 and induced CDX2 transcription. Our study for the first time revealed that CDX2 is directly regulated by FXR via transcriptional activation. Intriguingly, Modica et al identified CDX2 as a positive regulator of FXR expression in intestinal cells [46], implying that there might exist CDX2-FXR reciprocal regulation in colorectal tumorigenesis. Collectively, our study identified FXR as a novel mediator of CDX2 expression.

Additionally, the combination of OCA plus GSK126 synergistically elevated CDX2 expression in colon cancer cells and their derived xenograft tumor, compared to the single drug. The elevation of CDX2 might be attributed the upregulation of FXR and the accelerative nuclear localization of FXR, as demonstrated in this study. Correspondingly, the combination treatment triggered CDX2 regulatory pathway with the retardation of phosphorylation of Akt and GSK-3β and nuclear translocation of β-Catenin [29]. The expression of p21, E-cadherin, and active caspase-3 were upregulated, and the expression of cyclin D1, c-Myc, Snail, and MMP-9 was downregulated upon the drug combination.

Monoclonal antibodies against epidermal growth factor receptor (EGFR) or vascular endothelial growth factor (VEGF) have been entered the therapeutic regimen of metastatic CRC treatment. However, the effectiveness of EGFR inhibitor is constrained by intrinsic drug resistance due to KRAS mutations [47]. Intriguingly, Chen et al. reported that combined treatment of β-elemene and cetuximab elicited a synergistic antitumor effect on KRAS mutant CRC cells by induction of ferroptosis [48]. Given that the critical role of c-Src kinase in the intricate mechanisms of resistance to EGFR inhibitor, c-Src inhibitors in combination with EGFR inhibitors are at the stage of pre-clinical phase [49]. Hence, combination therapy based on monoclonal antibodies might offers an innovative and effective strategy for CRC patients with RAS mutations. The vital role of EZH2 in tumorigenesis highlight targeting EZH2 as a promising therapeutic strategy in cancer treatment. Multiple kinds of EZH2 inhibitor have been developed, and several clinical trials of target EZH2 for different cancer types are ongoing. GSK126 is a highly selective and potent inhibitor of EZH2. Recently, a phase I study revealed a modest anticancer effect of GSK126 on human hematologic and solid tumors, despite that drug administration and relatively short half-life limited effective exposure [50]. Combining EZH2 inhibitors with target therapy might improve the treatment efficacy and overcome the limitation of monotherapy. Co-inhibition of both EZH2 and EGFR elicited a synergic effect on tumor growth suppression in CRC by triggering autophagy and inducing apoptosis [51]. Our previous study revealed that that colon cancer cells exhibited the varying responses to OCA [16]. Herein, we identified the EZH2 inhibitor GSK126 with synergism with FXR agonist OCA in confronting CRC. The combination treatment thoroughly inhibited the aggressive phenotype of the four colon cancer cells by a series of in vitro and in vivo experiments. The combination treatment overcame the varied responses of four colon cancer cells to OCA. More importantly, the combined treatment had low toxic side effects for normal epithelial cells. Mechanistically, our study revealed that this synergistic effect was probably attributed to the acceleration of FXR nuclear location and the upregulation of CDX2 expression. However, the therapeutic effects of combination therapy require further evaluation in clinical trials, and the side effects and patient tolerance might limit the effectiveness.

Collectively, the present study for the first time demonstrated that the expression of FXR was positively correlated with EZH2 in colon cancer tissues. EZH2 transcriptionally suppressed FXR via H3K27me3, which subsequently activated the expression of tumor suppressor CDX2. The combination of FXR agonist plus EZH2 inhibitor exerts synergistic tumor inhibition in CRC both in vitro and in vivo by dramatically accelerating FXR nuclear location and cooperatively upregulating CDX2 expression. Our study offers useful evidence for the clinical use of FXR agonists combined with EZH2 inhibitors in combating CRC.

## Supplementary information

Uncropped western blot

cddis-author-contribution-form

aj-checklist

Supplementary legend

Supplementary table

Supplementary figure 1

Supplementary figure 2

Supplementary figure 3

Supplementary figure 4

## Data Availability

All data needed to evaluate the conclusions in the paper are present in the paper. Additional data related to this paper may be requested from the corresponding author.
