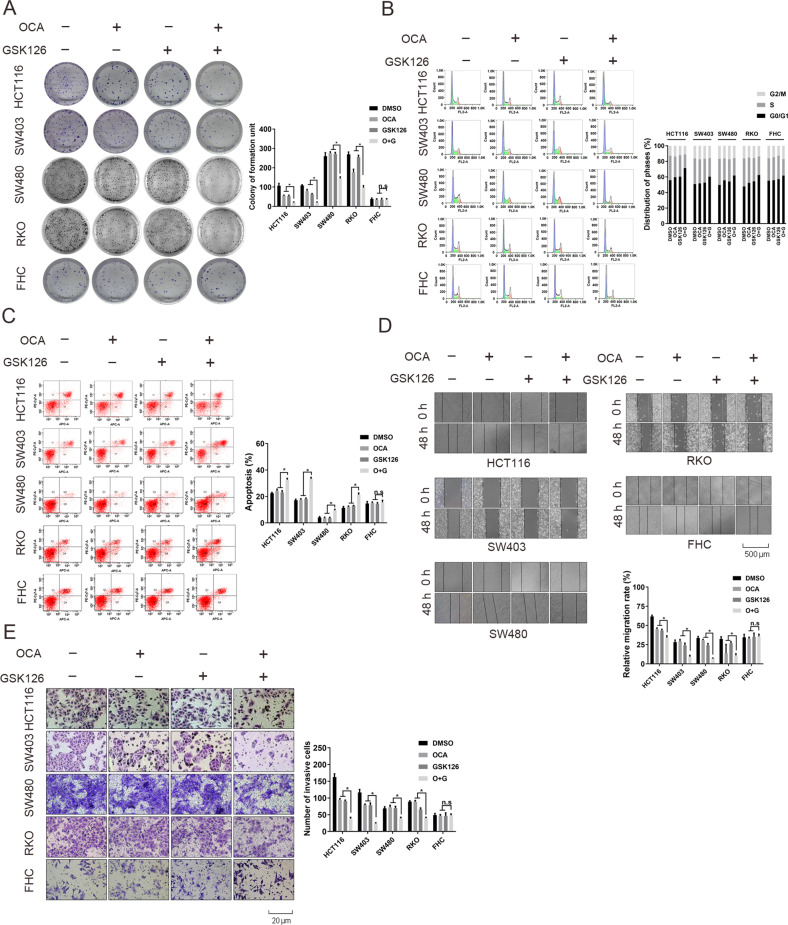# Correction: Activation of FXR and inhibition of EZH2 synergistically inhibit colorectal cancer through cooperatively accelerating FXR nuclear location and upregulating CDX2 expression

**DOI:** 10.1038/s41419-023-05662-x

**Published:** 2023-02-10

**Authors:** Junhui Yu, Kui Yang, Jianbao Zheng, Pengwei Zhao, Jie Xia, Xuejun Sun, Wei Zhao

**Affiliations:** 1 Department of General Surgery, First Affiliated Hospital of Xi’an Jiaotong University, 710061 Xi’an, PR China; 2 State Key Laboratory of Bioactive Substance and Function of Natural Medicines, Department of New Drug Research and Development, Institute of Materia Medica, Chinese Academy of Medical Sciences and Peking Union Medical College, 100050 Beijing, PR China

**Keywords:** Colon cancer, Target identification, Targeted therapies

Correction to: *Cell Death and Disease* 10.1038/s41419-022-04745-5, published online 21 April 2022

The original version of this article unfortunately contained a mistake in Figure 3E. The correct figure can be found below. The authors apologise for any inconvenience caused and have confirmed that our statistical results are basically unaffected. The original article has been corrected.