# Supplementary material for: Activation of FXR and inhibition of EZH2 synergistically inhibit colorectal cancer through cooperatively accelerating FXR nuclear location and upregulating CDX2 expression
Source: Cell Death Dis. 2022 Apr 21;13(4):388. doi: 10.1038/s41419-022-04745-5 (PMC9023572; doi:10.1038/s41419-022-04745-5)

Fig.1c

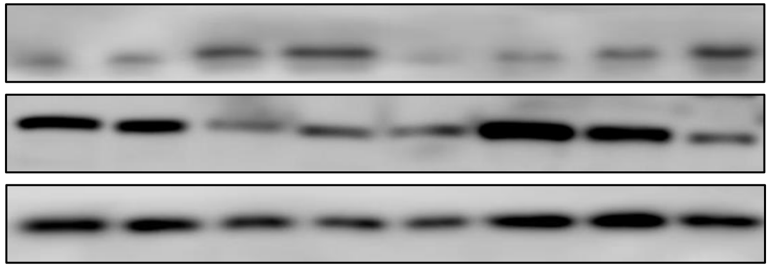

Fig.1c

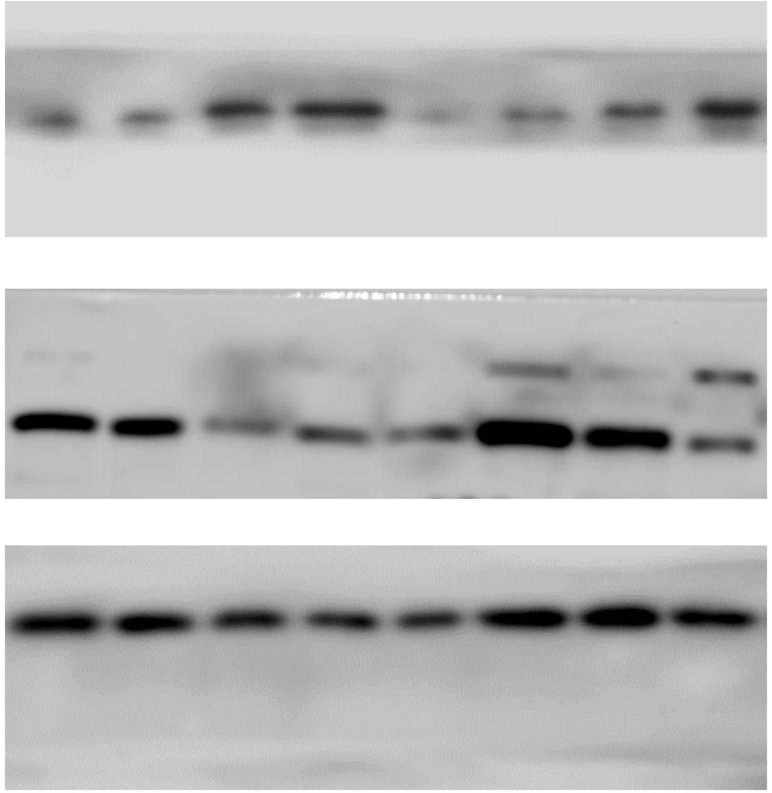

Fig.1d

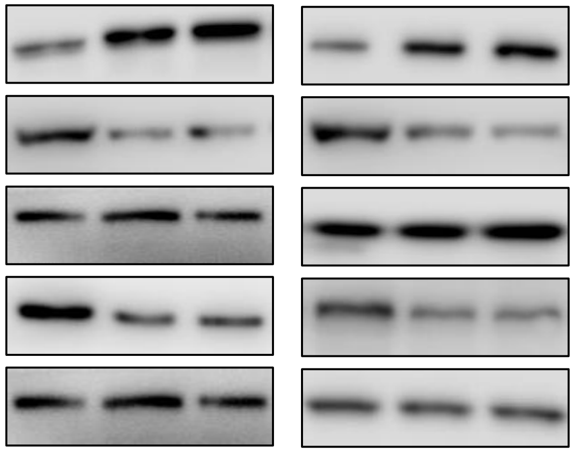

Fig.1d

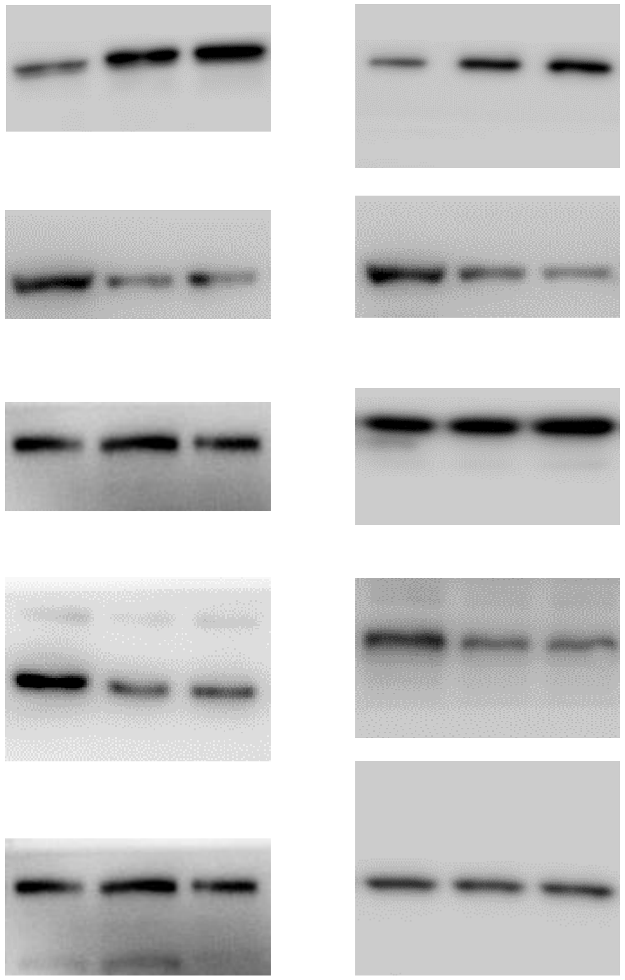

Fig.1e

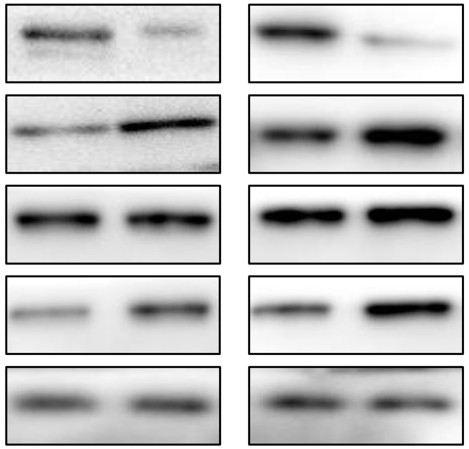

Fig.1e

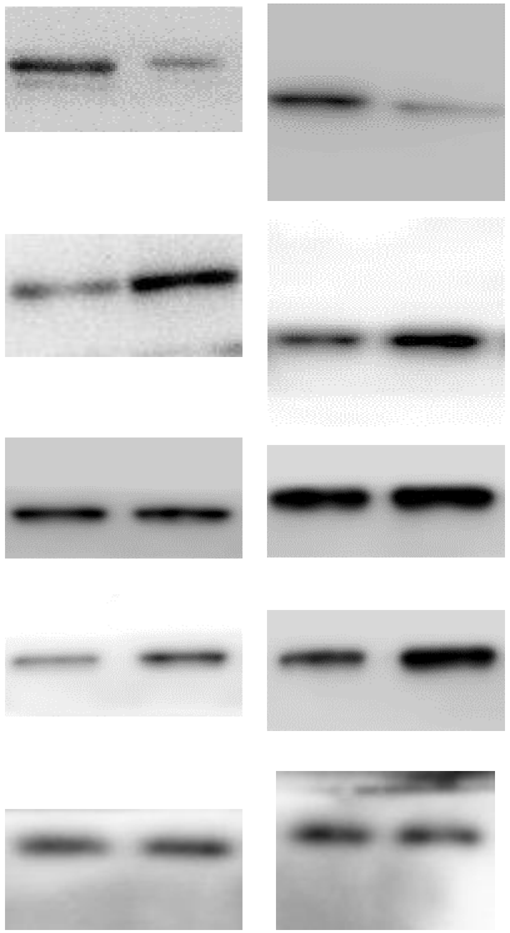

Fig.1f

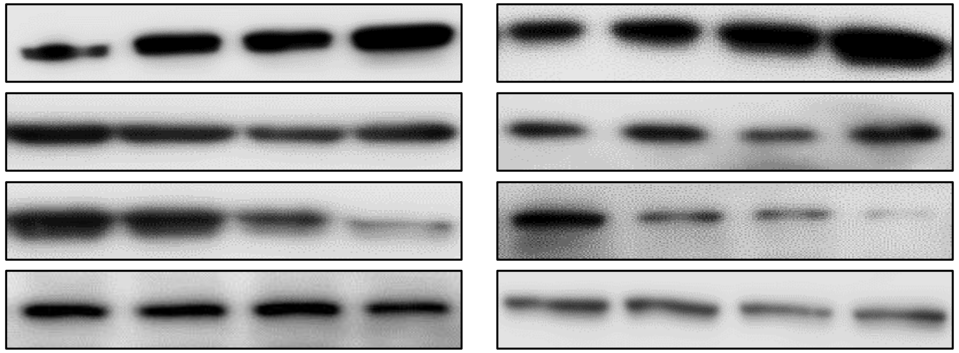

Fig.1f

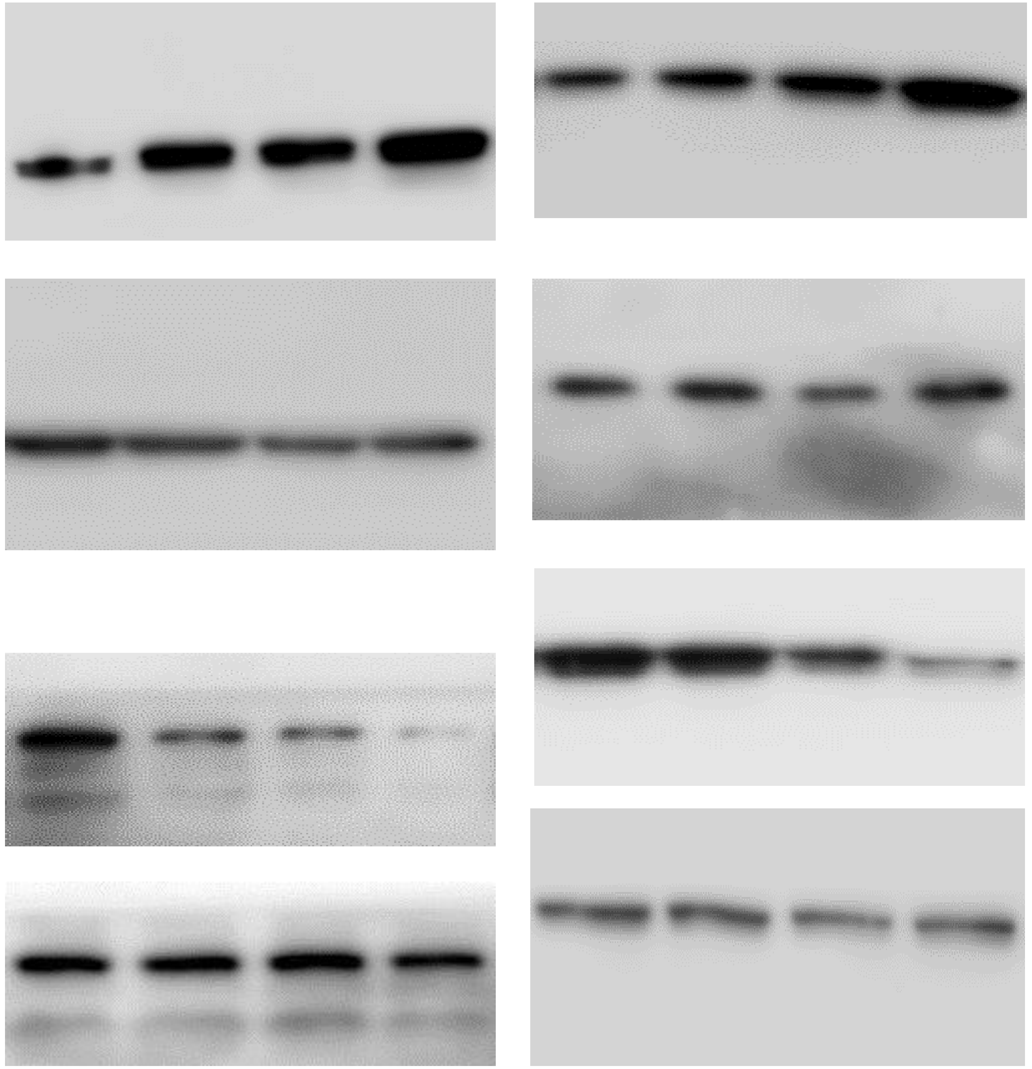

Fig.4a

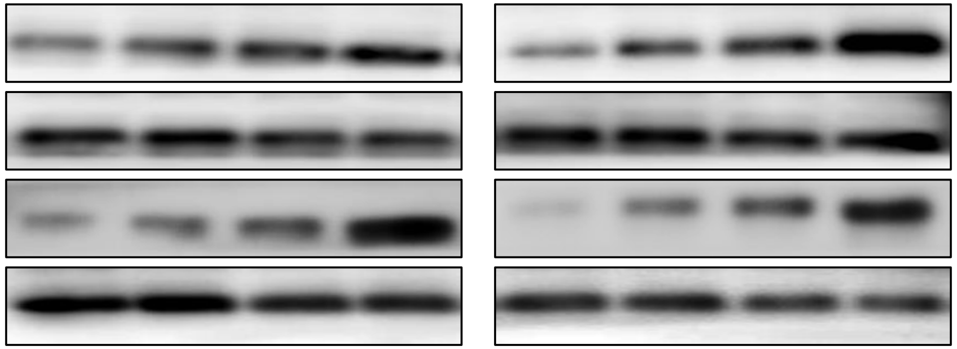

Fig.4a

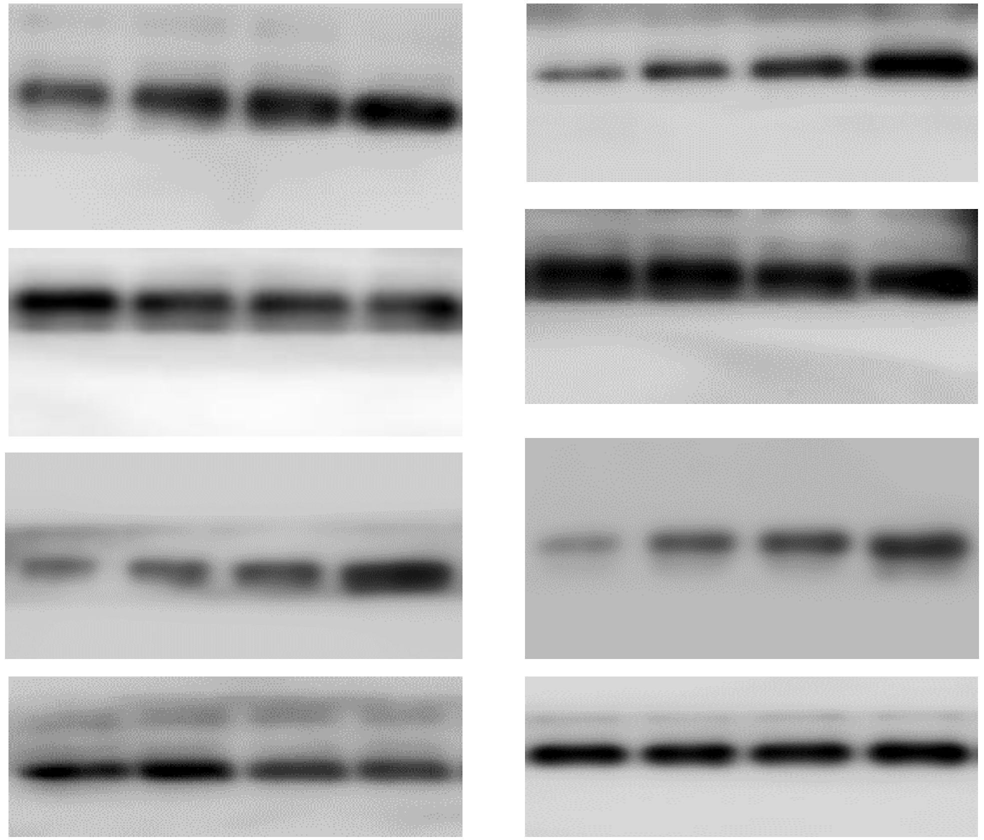

Fig.5a

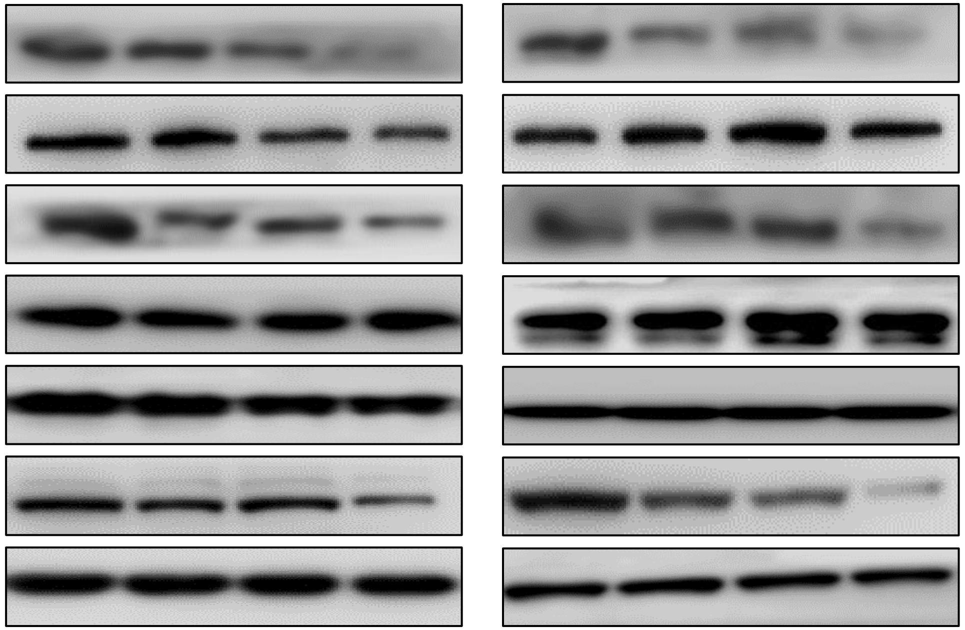

Fig.5a

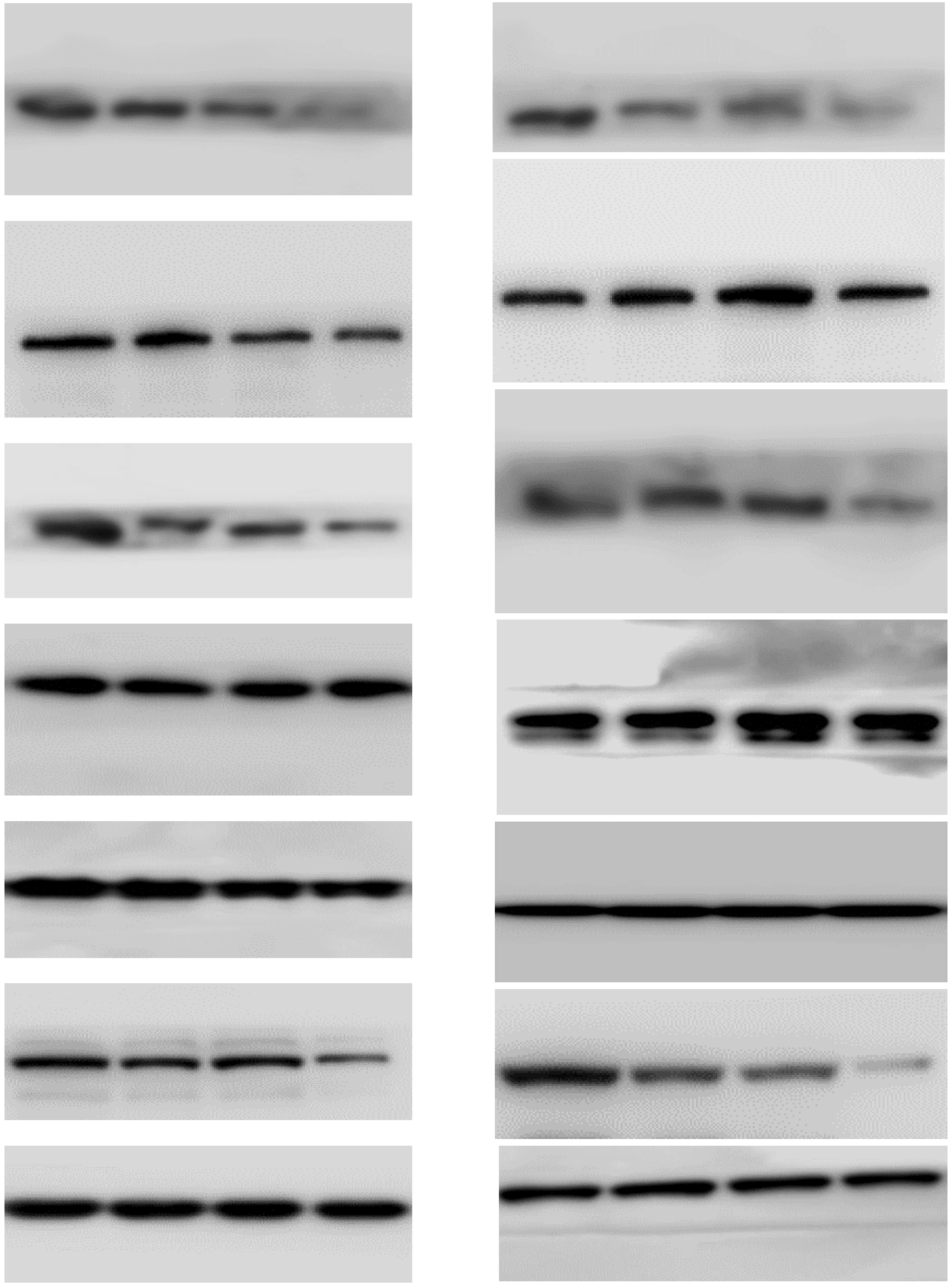

Fig.5b

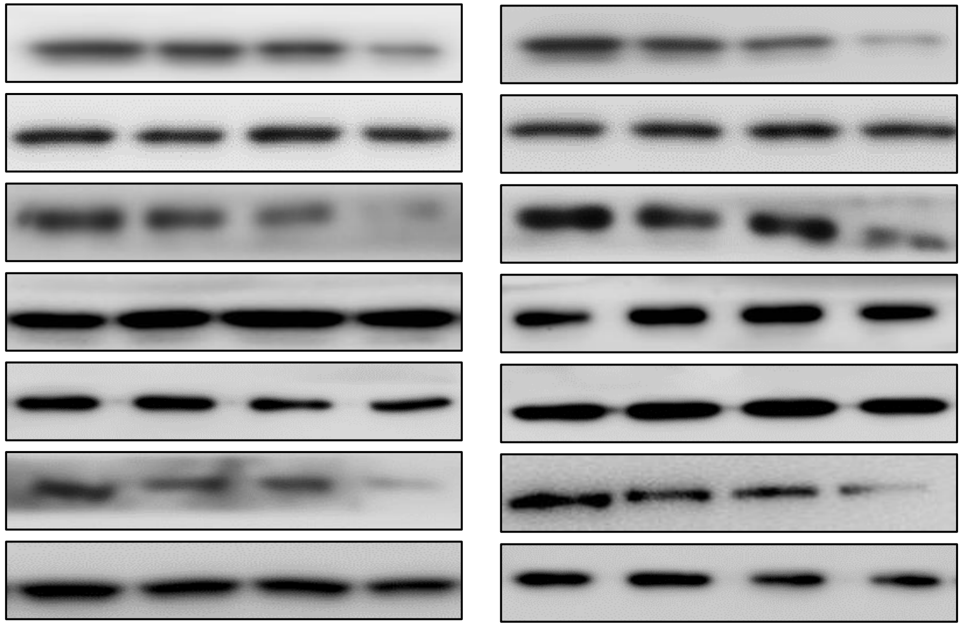

Fig.5b

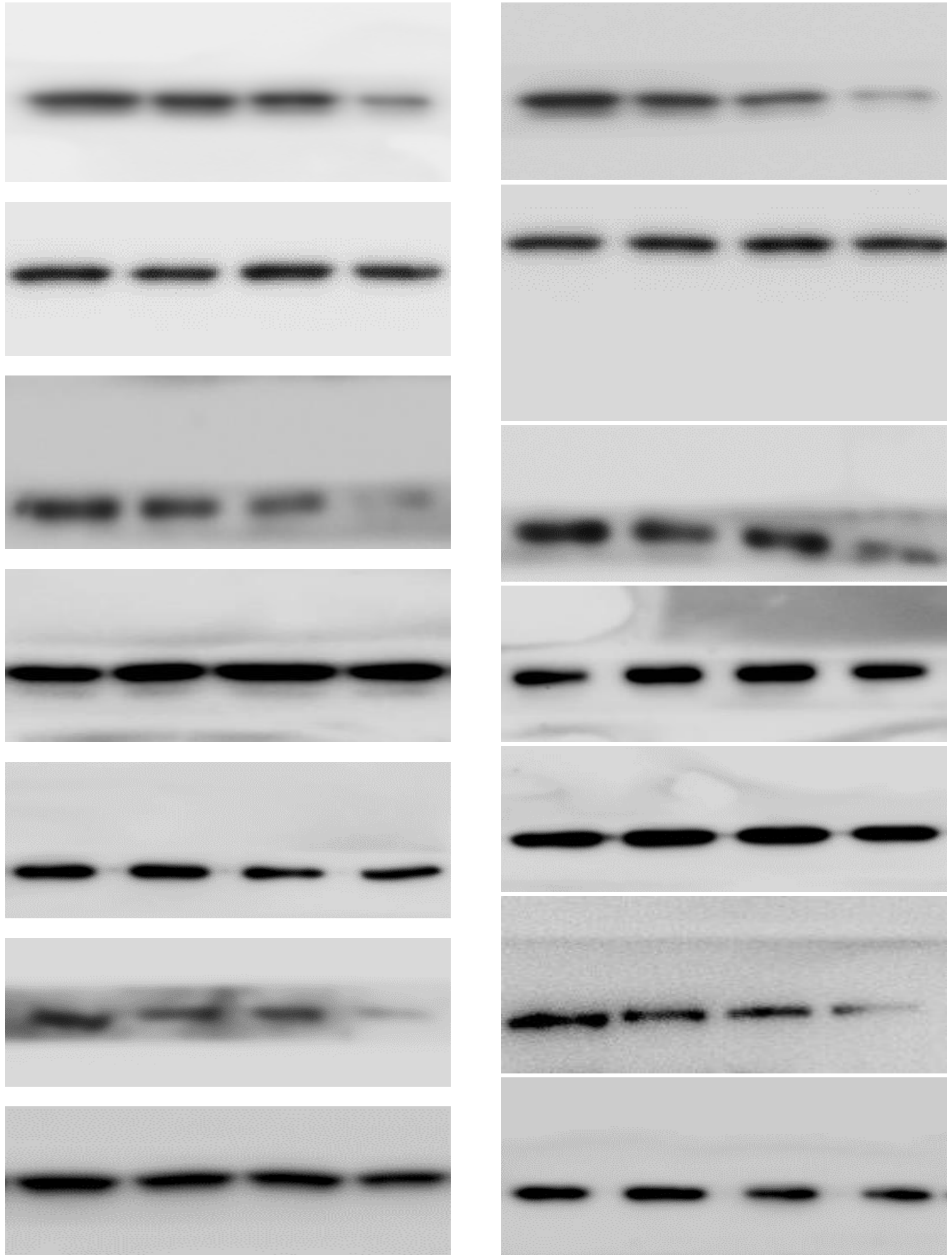

Fig.5c

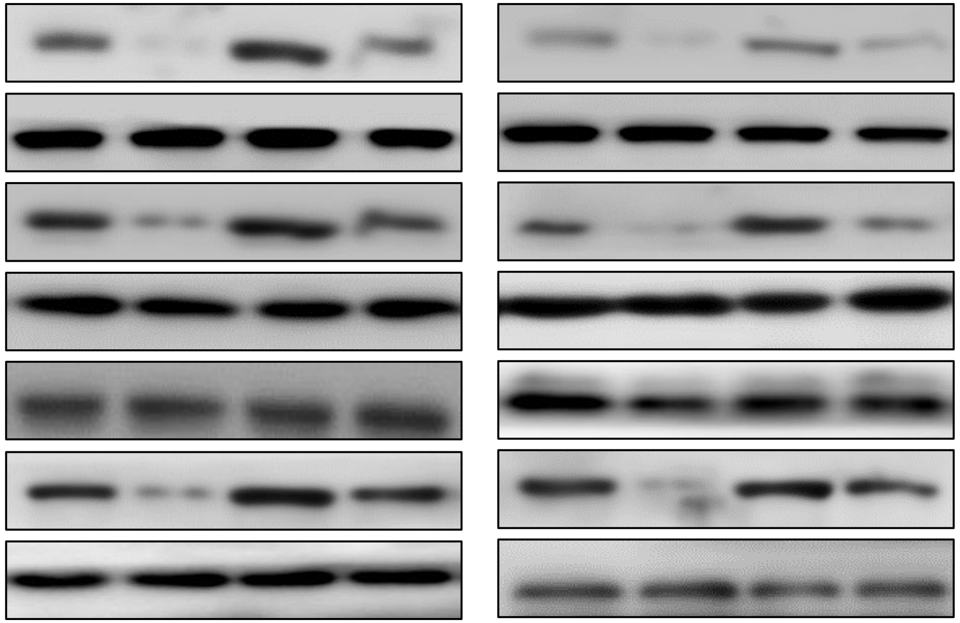

Fig.5c

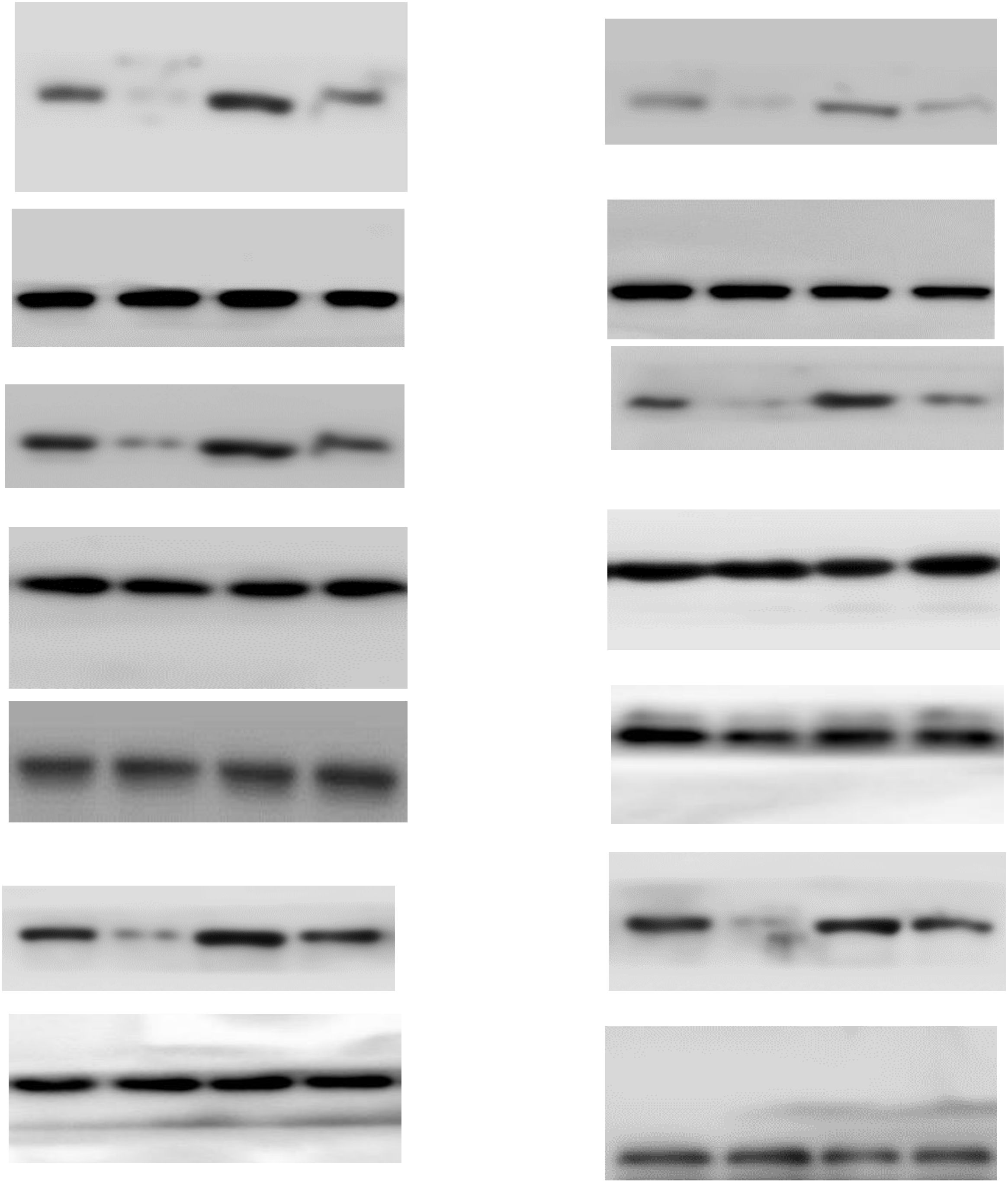

Fig.5d

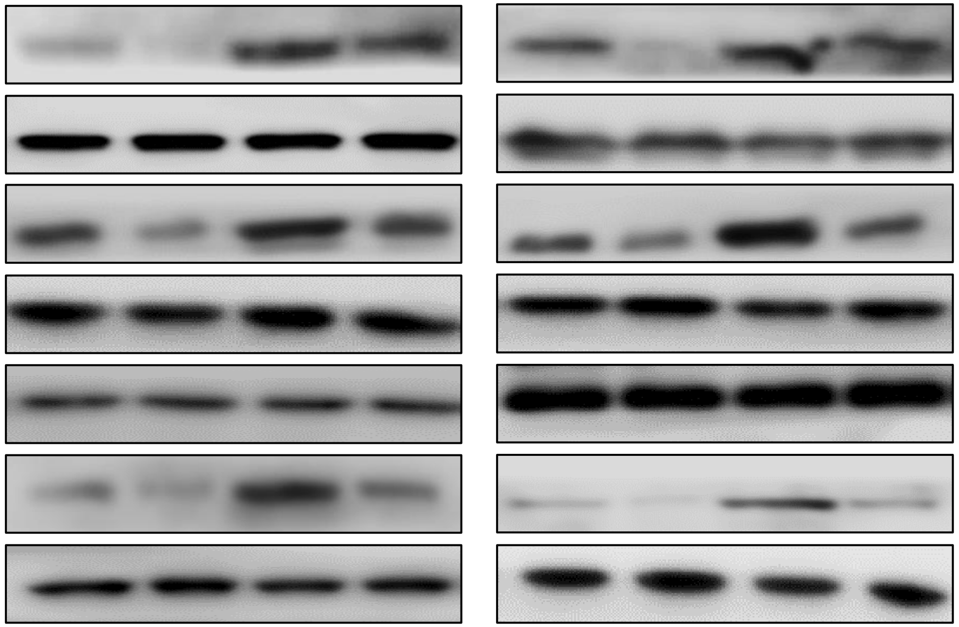

Fig.5d

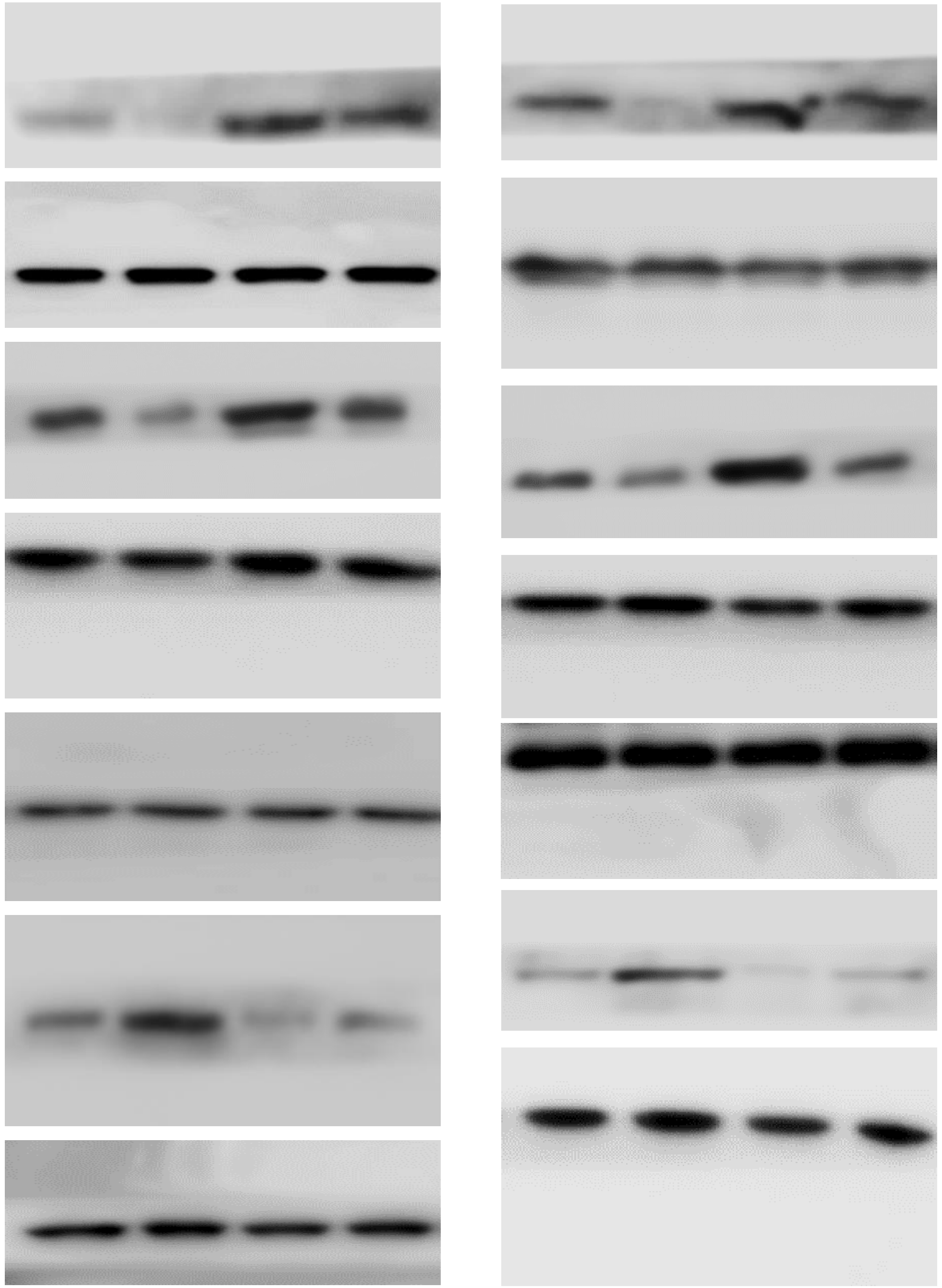

Fig.5e

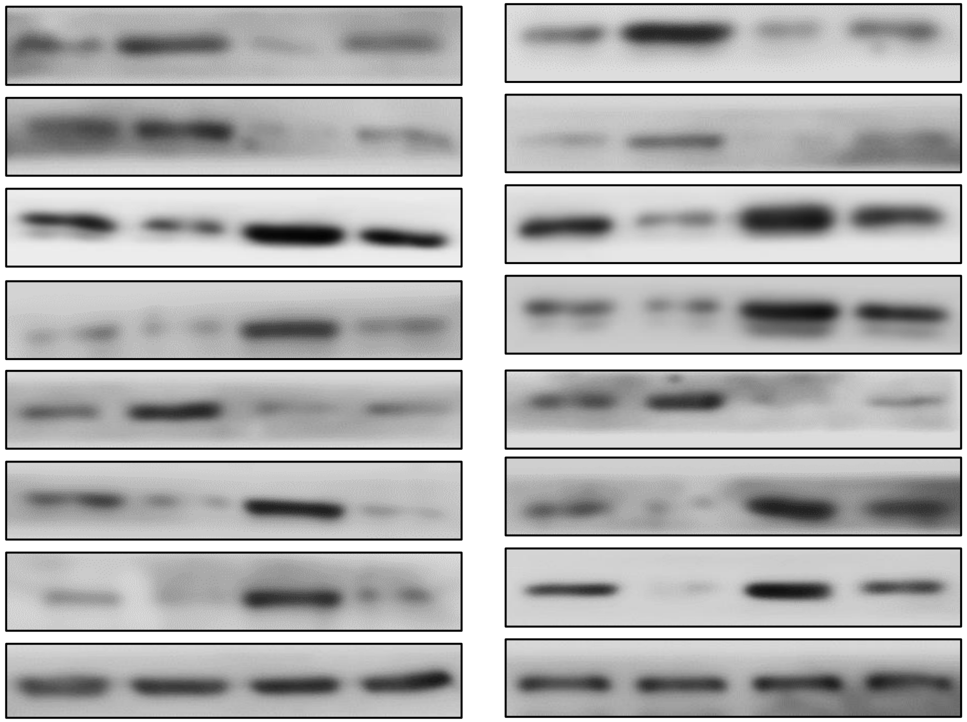

Fig.5e

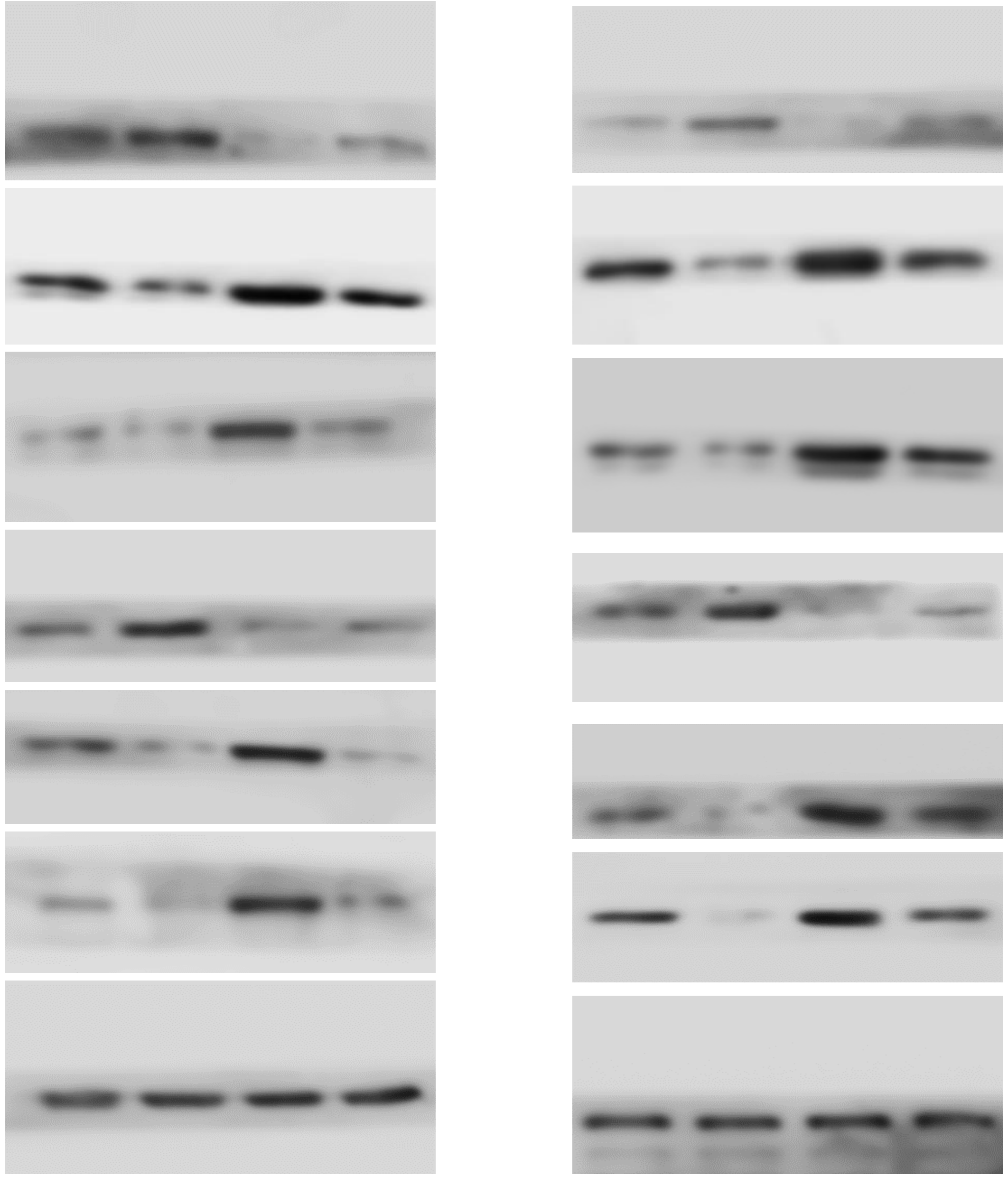

Supplementary Fig.1e

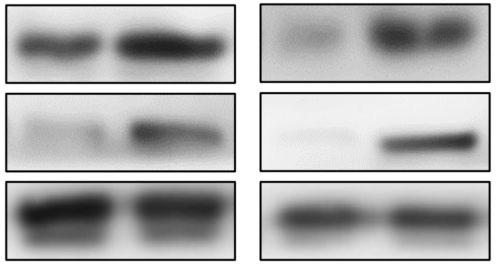

Supplementary Fig.1e

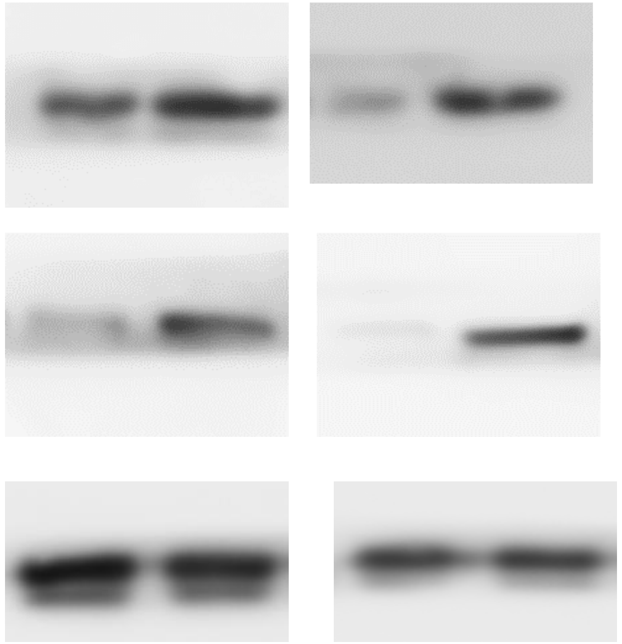

Supplementary Fig.1g

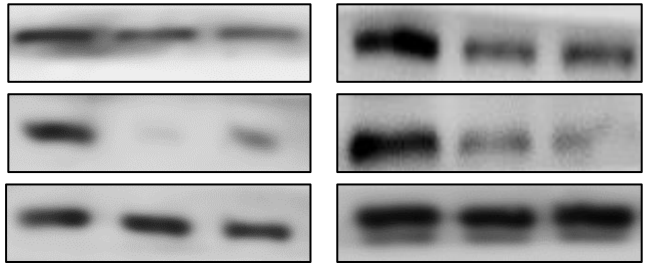

Supplementary Fig.1g

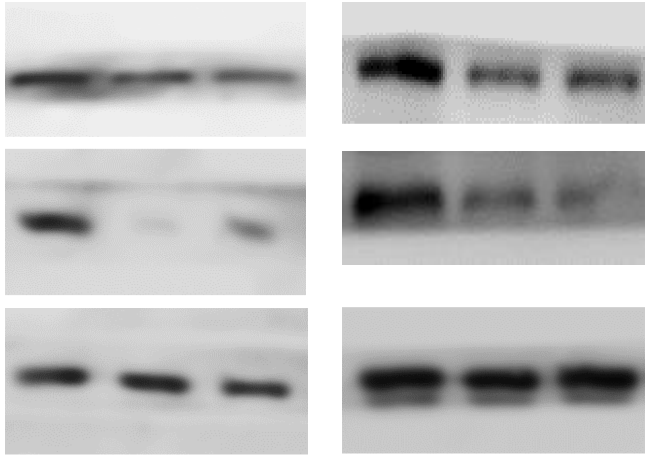

Supplementary Fig.3a

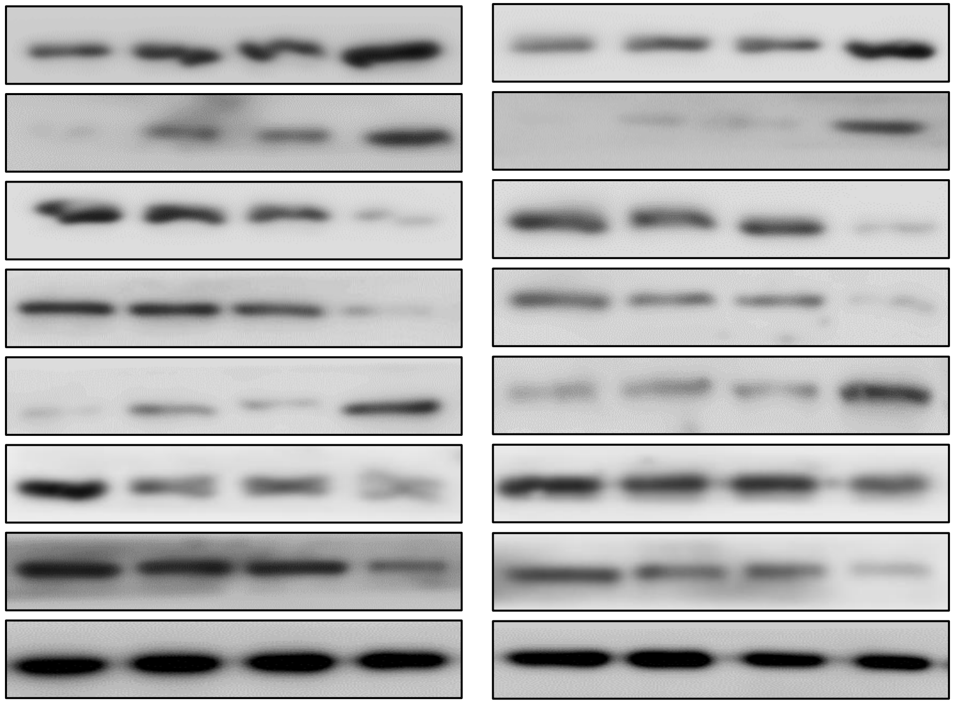

Supplementary Fig.3a

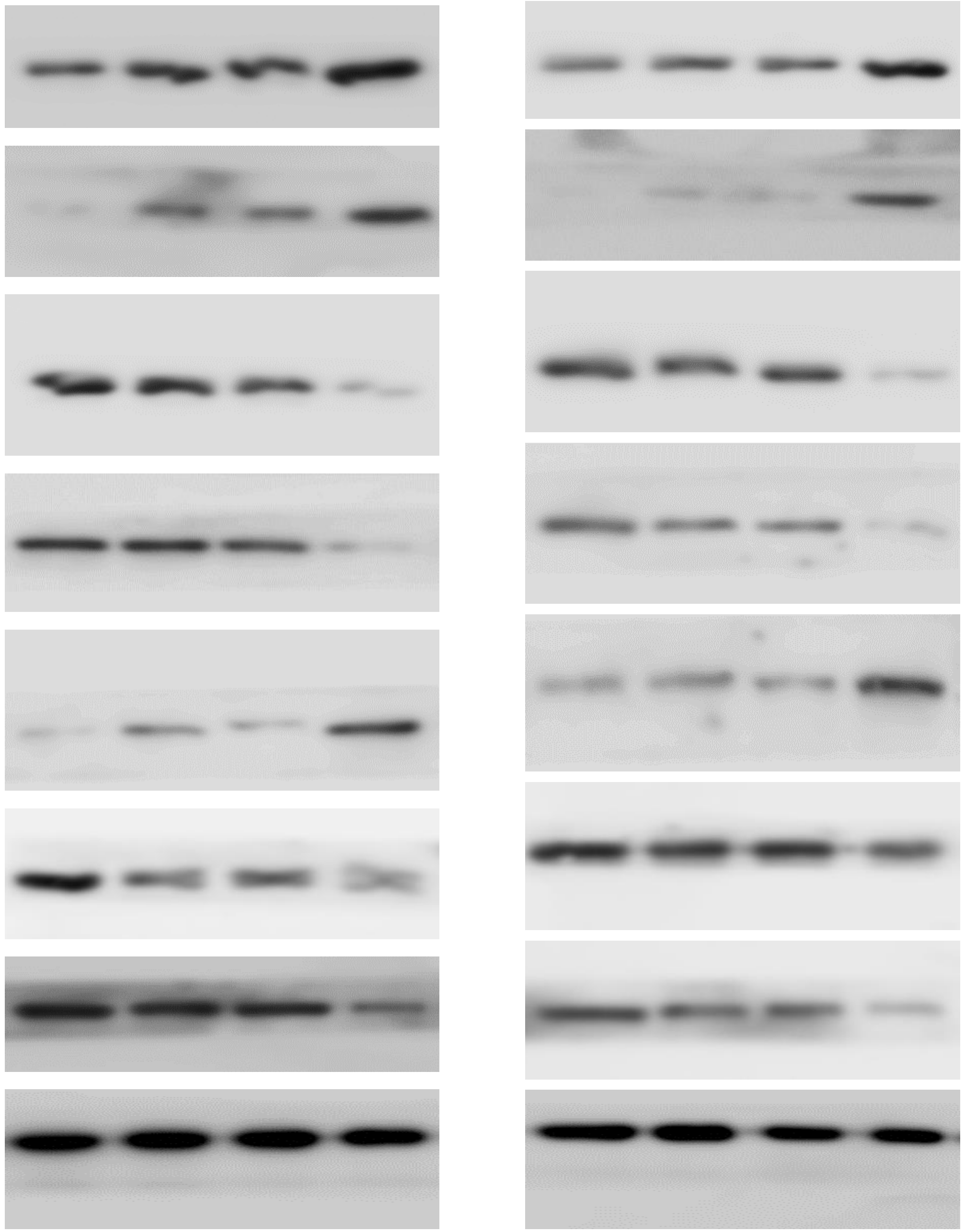

Supplementary Fig.3b

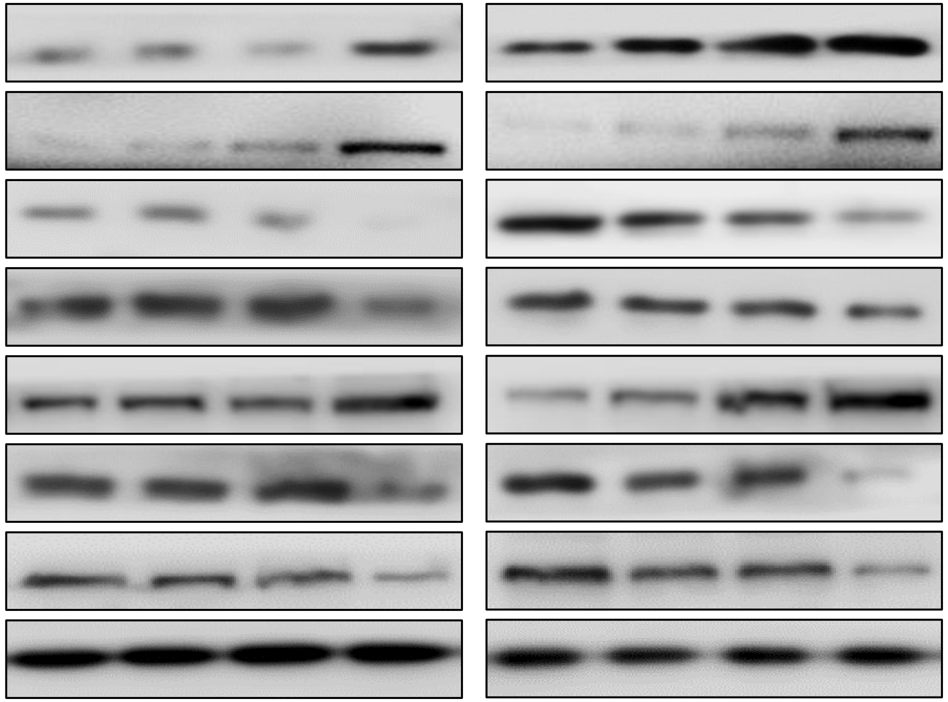

Supplementary Fig.3b

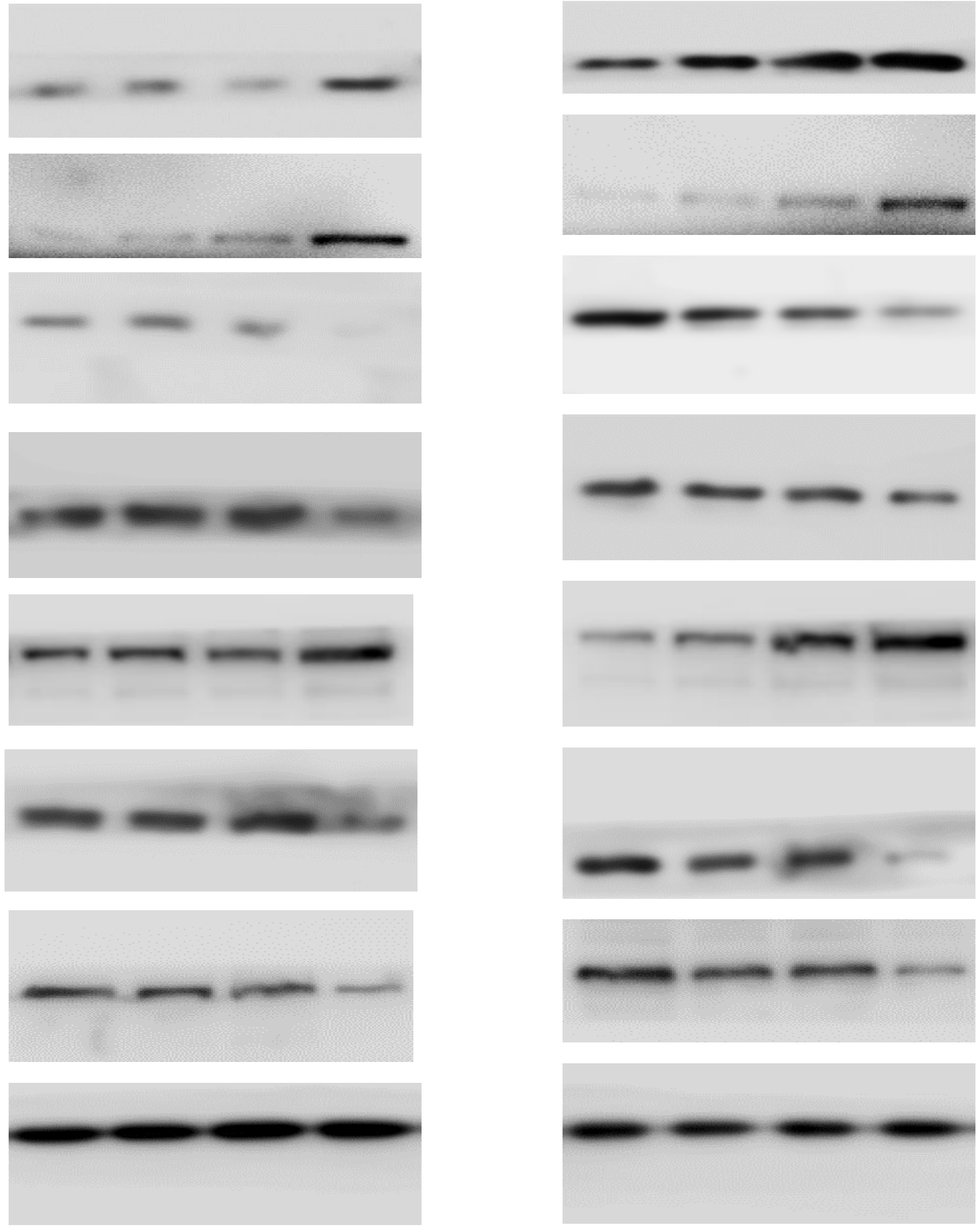

Supplement: Supplementary file 1 — Uncropped western blot [file 41419_2022_4745_MOESM1_ESM.pdf]
