# Supplementary material for: Activation of FXR and inhibition of EZH2 synergistically inhibit colorectal cancer through cooperatively accelerating FXR nuclear location and upregulating CDX2 expression
Source: Cell Death Dis. 2022 Apr 21;13(4):388. doi: 10.1038/s41419-022-04745-5 (PMC9023572; doi:10.1038/s41419-022-04745-5)
Supplement: Supplementary file 4 — Supplementary legend [file 41419_2022_4745_MOESM4_ESM.docx]

Supplementary figure 1 FXR transcriptionally activated the expression of tumor suppressor CDX2 **a** The activities of the CDX2 full promoter reporter construct and the truncated ones in FXR-overexpressing HCT116 and SW480 cells using the dual luciferase assay. **b** Schematic representation of the FXR putative binding sites (IR-1) in CDX2 promoter region (up panel). Enrichment level of FXR binding putative site in the CDX2 promoter region in HCT116 and SW480 cells was determined by qChIP assay (down panel). **c** The enrichment level of FXR to the IR-1 element in the CDX2 promoter region in HCT116 and SW480 cells treated with OCA and GSK126 alone or in combination by qChIP assay. **d, e** The mRNA (**d**) and protein (**e**, left panel: gel bands; right panel: quantitative analysis of these proteins) levels of FXR and CDX2 in FXR-overexpressing SW403 and SW480 cells. **f, g** The mRNA (**f**) and protein (**g**, left panel: gel bands; right panel: quantitative analysis of these proteins) levels of FXR and CDX2 in FXR-depleted HCT116 and RKO cells. All data are presented as the mean±SD from three independent experiments. **P* <0.05.

Supplementary figure 2 Drug combination screen identified GSK126 acting synergistically with OCA in colon epithelial cells. **a** The effect of FXR agonist OCA (left panel) and EZH2 inhibitor GSK126 (right panel) on the viability of colon epithelial cells FHC, HCT116, RKO, SW403, and SW480 detected by CCK8 assays. **b-f** Sensitivity of FHC (**b**), HCT116 (**c**), RKO (**d**), SW403 (**e**), and SW480 (**f**) to OCA, GSK126 alone, or OCA plus GSK126. Survival fraction (left) and the CI (right) are shown for each of these four cell lines. Fa fraction affected. Error bars represent means ± SD.

Supplementary figure 3 OCA and GSK126 synergistically inhibited the expression of cell cycle-, apoptosis-, and EMT-related proteins in colon cancer cells. **a** The protein levels of cell cycle-, apoptosis-, and EMT-related proteins in HCT116 and SW403 cells treated with OCA and GSK126 alone or in combination by western blotting analysis (left panel: gel bands; right panel: quantitative analysis of these proteins). **b** The protein levels of cell cycle-, apoptosis-, and EMT-related proteins in SW480 and RKO cells treated with OCA and GSK126 alone or in combination by western blotting analysis (left panel: gel bands; right panel: quantitative analysis of these proteins). All data are the mean ± SD of three independent experiments. **P* < 0.05.

Supplementary figure 4 OCA and GSK126 synergistically inhibited the activity of the Wnt/β-catenin pathway **a** The TOP/FOP-Flash reporter activities in HCT116 and SW480 cells with OCA and GSK126 alone or in combination. **b** The TOP/FOP-Flash reporter activities in HCT116 and SW480 cells with FXR overexpression, EZH2 depletion alone or in combination. **c** The effect of CDX2 depletion on the TOP/FOP-Flash reporter activities in HCT116 and SW480 cells with OCA and GSK126 alone or in combination. **d** The effect of CDX2 depletion on the TOP/FOP-Flash reporter activities in HCT116 and SW480 cells with FXR overexpression, EZH2 depletion alone or in combination. All data are the mean ± SD of three independent experiments. **P* < 0.05.
