# Supplementary material for: Activation of FXR and inhibition of EZH2 synergistically inhibit colorectal cancer through cooperatively accelerating FXR nuclear location and upregulating CDX2 expression
Source: Cell Death Dis. 2022 Apr 21;13(4):388. doi: 10.1038/s41419-022-04745-5 (PMC9023572; doi:10.1038/s41419-022-04745-5)
Supplement: Supplementary file 5 — Supplementary table [file 41419_2022_4745_MOESM5_ESM.docx]

Supplementary Table 1 Primer sequence

| Gene | Sequence |
| --- | --- |
| RT-PCR | |
| CDX2 | F: 5’-GAACCTGTGCGAGTGGATG-3’ |
|  | R: 5’-GGATGGTGATGTAGCGACTG-3’ |
| FXR | F: 5’-TGCAGATCAGACCGTGAATGA-3’ |
|  | R: 5’-TTGGTTGCCATTTCCGTCAAA-3’ |
| GAPDH | F: 5’-TGCACCACCAACTGCTTAGC-3’ |
|  | R: 5’-GGCATGGACTGTGGTCATGAG-3’ |
| Luciferase Assays |  |
| F1(-1059 bp **-** +89 bp) | F: 5’-CGCCTCCAGTTATGCACGCCA-3’ |
| F2(-819 bp **-** +89 bp) | F: 5’-TTTGGGGCTGCAGTCGTCCG-3’ |
| F3(-519 bp **-** +89 bp) | F: 5’-GCACCAGGTTGGAAGGAGGAAGC-3’ |
| F4(-376 bp **-** +89 bp) | F: 5’-CTGCGCCTCGACGTCTCCAA-3’ |
| F5(-157bp **-** +89 bp) | F: 5’-AGAAGAGCCGCGAGGAGCCA-3’ |
|  | R: 5’-TTCTGCGGCGCCAGGTTGAG-3’ |
| ChIP-qPCR  IR-1(-519 bp **-** -357 bp) | F: 5’-GCACCAGGTTGGAAGGAGGAAGC -3’ |
|  | R: 5’- TTGGAGACGTCGAGGCGCAG-3’ |
|  | F: 5’-TGACCTAGCAACCTGACCAG-3’ |
| 3’-UTR | F: 5’-CACAGGGGCGCCTGAGGTTG-3’ |
|  | F: 5’-GGGTTCACGAGCCCAAGCCC-3’ |

Supplemental Table 2 Antibodies

| Antibodies | Source | | Identifier |
| --- | --- | --- | --- |
| GAPDH | | Santa Cruz | Cat#sc-47724 |
| FXR | | Santa Cruz | Cat#sc-25309 |
| EZH2 | | Abcam | Cat#ab191250 |
| SHP | | Abcam | Cat#ab96605 |
| CASP-3 | | Abcam | Cat#ab13847 |
| MMP-9 | | Abcam | Cat#ab76003 |
| Snail | | Abcam | Cat#ab53519 |
| p-AKT^Ser473^ | | Abcam | Cat#ab81283 |
| AKT | | Abcam | Cat#ab8805 |
| GSK-3β | | Cell Signaling Technology | Cat#12456 |
| GSK-3β ^Ser9^ | | Cell Signaling Technology | Cat#5558 |
| CDX2 | | Cell Signaling Technology | Cat#12306 |
| E-cadherin | | Cell Signaling Technology | Cat#14472 |
| cyclin D1 | | Cell Signaling Technology | Cat#55506 |
| c-Myc | | Cell Signaling Technology | Cat#18583 |
| p21^CIP1^ | | Cell Signaling Technology | Cat#2947 |
| β-catenin | | Cell Signaling Technology | Cat#8480 |
| Histone H3 | | Cell Signaling Technology | Cat#4499 |
