# Supplementary figures and images for: Activation of FXR and inhibition of EZH2 synergistically inhibit colorectal cancer through cooperatively accelerating FXR nuclear location and upregulating CDX2 expression
Source: Cell Death Dis. 2022 Apr 21;13(4):388. doi: 10.1038/s41419-022-04745-5 (PMC9023572; doi:10.1038/s41419-022-04745-5)

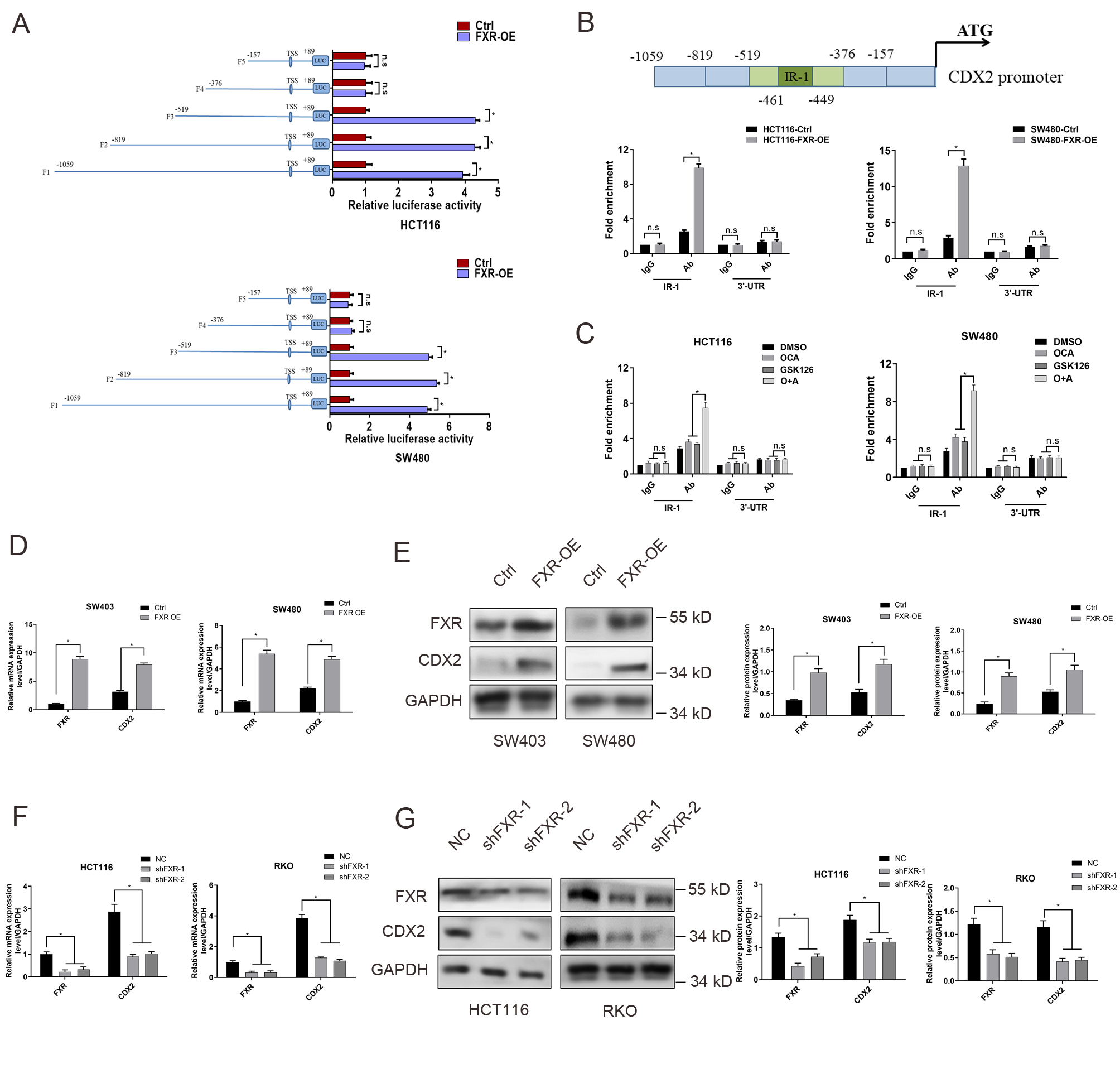

Supplement: Supplementary file 6 — Supplementary figure 1 [file 41419_2022_4745_MOESM6_ESM.tif]

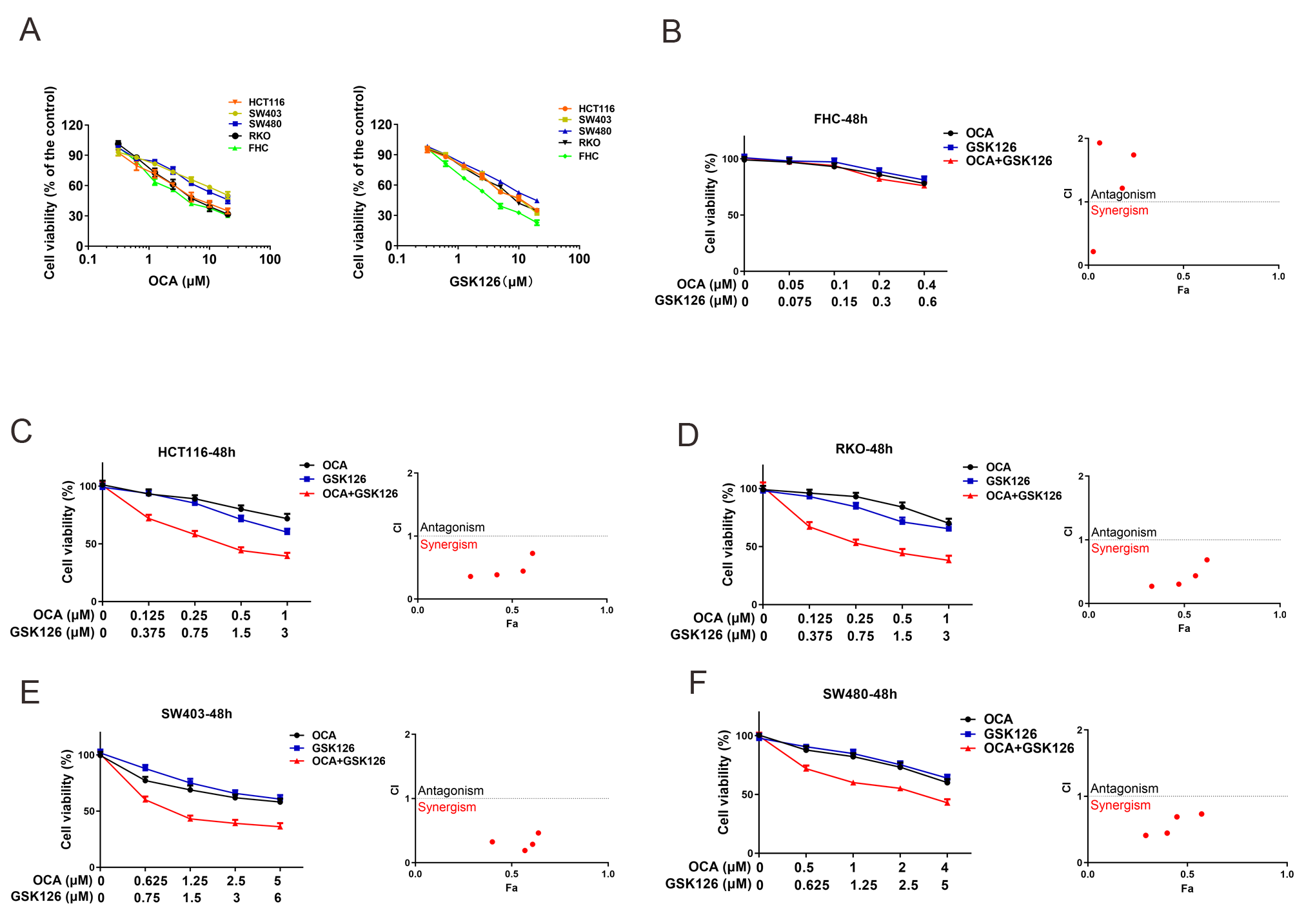

Supplement: Supplementary file 7 — Supplementary figure 2 [file 41419_2022_4745_MOESM7_ESM.tif]

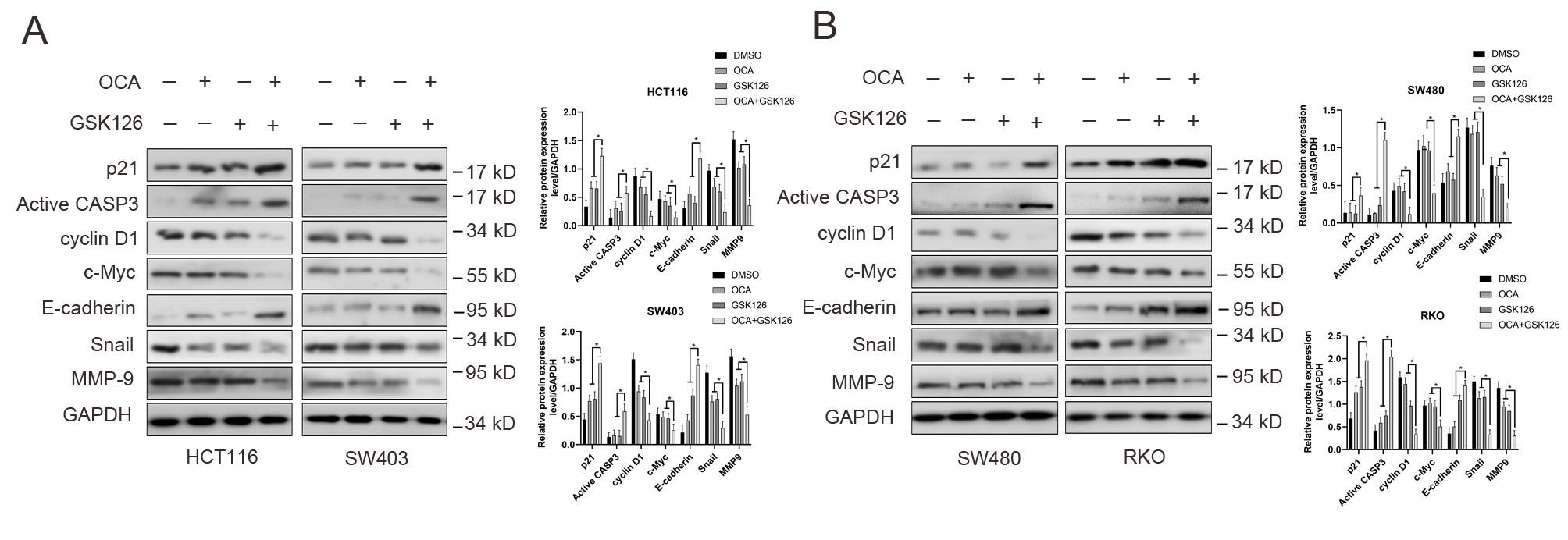

Supplement: Supplementary file 8 — Supplementary figure 3 [file 41419_2022_4745_MOESM8_ESM.tif]

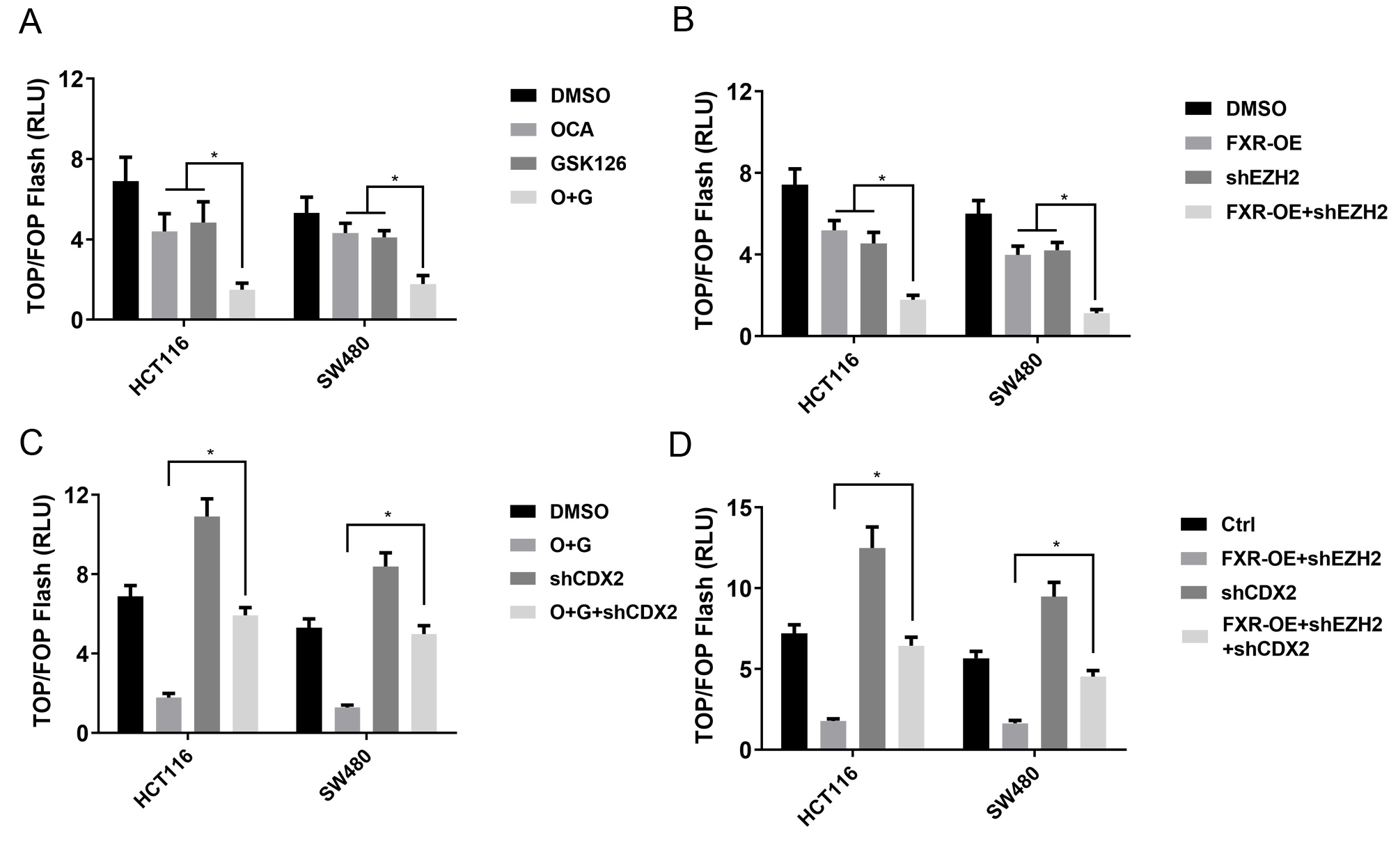

Supplement: Supplementary file 9 — Supplementary figure 4 [file 41419_2022_4745_MOESM9_ESM.tif]
